# *Paralia* (Bacillariophyta) stowaways in ship ballast: implications for biogeography and diversity of the genus

**DOI:** 10.1186/s40709-015-0024-5

**Published:** 2015-02-15

**Authors:** Michael L MacGillivary, Irena Kaczmarska

**Affiliations:** Department of Biology, Mount Allison University, 63B York Street, Sackville, NB E4L 1G7 Canada

**Keywords:** Diatoms, Lectotype, Epitype, Molecular taxonomy, *Paralia* species complex, Cryptic diversity, ITS2 secondary structure, Ship ballast

## Abstract

**Background:**

The genus *Paralia* Heiberg is one of the most recognizable, widely distributed and commonly reported diatoms from contemporary coastal marine environments and ship ballast. Species discovery has historically been made in diatoms through the recognition of morphological discontinuities between specimens, first using light and later electron microscopy. However, recently, morphologically semi-cryptic species of *Paralia* were delineated using genetic analyses, among mostly tropical and subtropical sites.

**Results:**

Ten morphological characters of the frustules and sequence fragments from the nuclear genome (conserved 18S regions of ribosomal RNA and the variable internal transcribed spacer [ITS]), and from the RuBisCo large subunit (*rbc*L) gene of the chloroplast genome were examined. Frustule morphology did not segregate species, however, comparisons of sequence fragments and ITS2 secondary structures yielded a new species from North American waters, *P. guyana* (with four genodemes), and another widely-distributed species, *P. marina*. The latter was lecto- and epitypified here because it is most similar to specimens in the type preparation BM1021 representing Smith’s concept of the species. *Paralia marina* and certain genodemes of *P. guyana* were morphologically cryptic. Only those genodemes of *P. guyana* that possess prickly separation valves could be morphologically distinguished from *P. marina* with relative confidence in SEM preparations. All clones established from chains isolated from the ballast sediment of the ships sailing along the Atlantic coast of North America belonged to *P. guyana*. All DNA sequences of preserved *Paralia* chains recovered from the three trans-Atlantic voyages (TAVs) samples arriving to eastern Canada from Europe shared 100% identity with *P. marina*.

**Conclusion:**

First, if the $\overline{x}$ = 130592 *P. marina* cells per ballast tank at the end of the TAVs represents their abundance in ballast tanks of similar crossings and following mid-ocean ballast water exchange, then this diatom, if de-ballasted, exerts a strong and continued propagule pressure on Eastern Canadian coasts. Despite this, as of 2009, *P. marina* was found only in Cheticamp, Nova Scotia, Canada. Second, genetic analysis readily segregated cryptic and semi-cryptic taxa of *Paralia*, highlighting the usefulness of the molecular approach to species recognition, e.g., in programs monitoring alien introductions.

**Electronic supplementary material:**

The online version of this article (doi:10.1186/s40709-015-0024-5) contains supplementary material, which is available to authorized users.

## Background

Evaluation of several cosmopolitan, eurytopic or taxonomically challenging diatom species from a wide spectrum of genera with a combination of comprehensive morphometric and molecular analyses has consistently led to the discovery of a number of new cryptic and semi-cryptic following [1,2] species e.g., [3-8]. However, these are a minute fraction of the 100000 or more extant diatom species [9]. Thus the application of systematic analysis using both morphometric and molecular approaches to previously less-explored taxa would likely result in the discovery of more new species.

The commonly reported, easily-recognisable, centric diatom *Paralia sulcata* (hereafter referred to as *P. sulcata sensu lato* [broadly defined or *s.l*.] to indicate reports published before *P. sulcata sensu stricto* [strictly defined or *s.s*.] was lectotypified [10]) is known worldwide from coastal marine waters and contemporary and fossil sediments. Such records may reflect a truly cosmopolitan nature of the species, but may also be an artefact of relying solely on Light Microscopy (LM) for species identification. LM is insufficient to resolve all diagnostic characters necessary to identify some of the species of *Paralia* [6,10-13]. For example, the use of Scanning Electron Microscopy (SEM) revealed a number of new *Paralia* species including: *P. elliptica* Garcia [14], *P. fenestrata* Sawai and Nagumo and *P. capitata* Sawai and Nagumo [12], *P. longispina* Konno and Jordan [13] and *P. ehrmanii* MacGillivary [6]. Two additional new species, *P. allisonii* MacGillivary and *P. crawfordii* MacGillivary, were delineated using both morphological and molecular characters [6]; the first two such cases in the genus. Nonetheless, the studies above do not represent a global survey of *Paralia* populations and the global species richness of this genus is not yet fully known.

More than one entity conforming to a historically practiced broad diagnosis of *Paralia sulcata* [15] has recently been discovered [10]. However, fossilised specimens of *Paralia* Heiberg from Ehrenberg’s original collection have since become available for SEM examination. This original gathering revealed the existence of two morphologically discrete taxa; *P. sulcata* (corresponding to Ehrenberg’s description [16]) and a new species, *P. obscura* MacGillivary. The latter is morphologically similar to *P. sulcata* (*sensu* [15]). Crawford [15] inferred that the Plymouth specimens of *Paralia* corresponded to Ehrenberg’s *P. sulcata* and were the same (pg. 209 of [15] as far as it could have been determined at that time) as those present on Smith’s BM1021 preparation (designated as the generitype slide of *Paralia* [15] labelled *Orthosira marina* [17]). Consequently, a better understanding of Smith’s [17] concept of *O. marina*, later transferred by Heiberg [18] to *Paralia* (*P. marina* (Smith) Heiberg), may render circumscription of this species more precisely and therefore better clarify its affiliation to Ehrenberg’s *P. sulcata*, about which Smith himself was somewhat unclear (pg. 60 in [17]).

*Paralia sulcata s.l*. is also one of the most common diatom-stowaways in ships’ ballast tanks [19-25]. Ship ballast is an important human-mediated transport vector for the unintentional introduction of non-native, potentially invasive species to new regions e.g., [19,26-28] and has been held responsible for the establishment of diatoms outside their native range, such as, *Coscinodiscus wailesii* Gran & Angst [29], *Odontella sinensis* (Greville) Grunow [30] or *Thalassiosira punctigera* (Castracane) Hasle [31].

The potential for non-native species introductions has grown in the past 50 years due to an increase in both the volume of ballast water carried by individual commercial vessels and the magnitude of international trade e.g., [26,32,33]. These developments have led international ports and their neighbourhoods to incur an overall higher propagule pressure of alien organisms which is, in part, a function of the number of individuals introduced and the rate at which they arrive [34-36]. Consequently, the number of ballast water mediated introductions of non-indigenous and even invasive species has continued to grow [37,38]. These introductions may remain underestimated for smaller organisms due to insufficient knowledge of native, regional florae that have not been systematically examined before industrial communities began to change their coastal environments. This is particularly true for microscopic organisms, including diatoms, where application of molecular means of species recognition [39-43] has demonstrated the presence of semi-cryptic or cryptic taxa, some of which survive in ship ballasts with days-long transport out of their native range [44].

The aims of this study were three-fold. First morphological (SEM-based) and molecular tools (18S, *rbc*L and ITS DNA sequences) were used to characterise 76 clones of *Paralia* isolated from the coastal waters of Canada, live sediments collected from ballast tanks of intercoastal ships docked at the ports of Saint John, New Brunswick and Halifax, Nova Scotia, and from a number of sites worldwide. Second, these results were compared to ITS DNA data recovered from the single-chain amplification of ethanol-fixed *Paralia* cells collected from ballast tank water in three trans-Atlantic vessels arriving to eastern Canadian ports from Europe to evaluate the potential for transport of European *Paralia* to Canada. Finally, specimens of *Paralia* from the preparation BM1021 were examined using LM in order to better understand Smith’s [17] concept of *P. marina* (as *Orthosira marina*) and to relate it to our clones.

## Results

### Species identity

**Figure 1:**
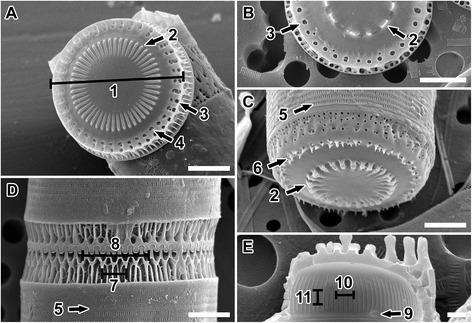
**SEM images illustrating the frustule characters examined and quantified in this study. (A)** intercalary valve face. 1 = diameter; 2 = internal linking spines (so named because they are located internally relative to the marginal linking spines) in the form of baculiform elevations in intercalary valves and as noduliform protrusions in separation valves; 3 = external ring of marginal pores; 4 = internal ring of marginal pores. **(B)** separation valve face. **(C)** Tilted view of separation valve. 5 = slits in copula; 6 = valve face prickles. **(D)** mantle. 7 = four fenestrae; 8 = six marginal linking spines. **(E)** Internal view of valve. 9 = rimoportulae; 10 = three internal striae; 11 = three internal striae pore areolae. **(A)** *Paralia guyana* ‘servidei’, **(B,E)** *P. guyana* ‘smooth’ and **(C,D)** *P. guyana* ‘capebreton’ genodemes. Scale bars = 5 μm **(A-D)**, 1 μm **(E)**.

**Figure 2:**
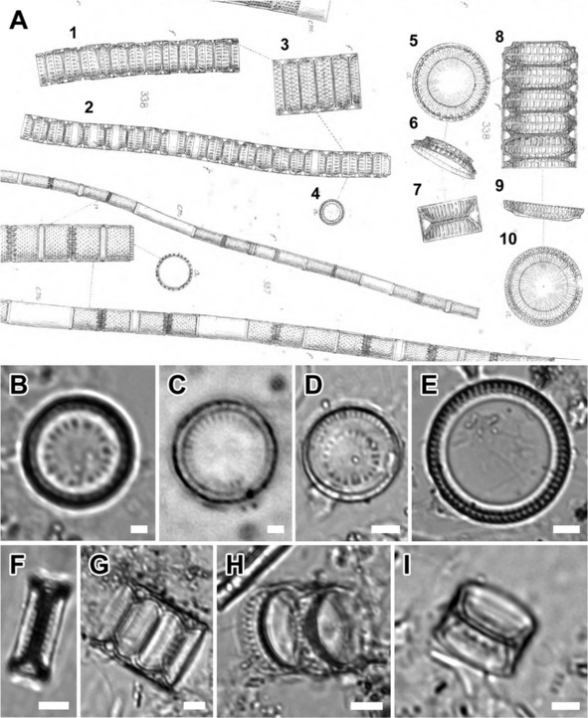
***Paralia*** **specimens from W. Smith’s ([** 17 **], pl. 53, figure 338) drawings and BM1021 preparation.** The BM1021 preparation, containing *P. marina* from Kinsale Harbour, Ireland (circa ca. 1850) is the generitype for *Paralia* [15]. Valves and chains labelled 1–4 and 7 correspond to Smith’s concept of *P. marina*, as deduced from BM1021 preparation. Those labelled 5, 6 and 8–10 show fenestrae which correspond more closely to *P. sulcata s.s*. [10] and *P. fenestrata* [12]. **(A)** Smith’s ([17], pl. 53, figure 338) drawings of valves and chains. **(B-D)** Valve face view. **(B,C)** Large and smaller intercalary valves. **(D)** Separation valve. **(E)** Internal view of valve. **(F-I)** Mantle of interlocked valves. **(F-H)** Intercalary. **(I)** Separation. Scale bars = 5 μm **(D-I)**, 2 μm **(B,C)**.

**Figure 3:**
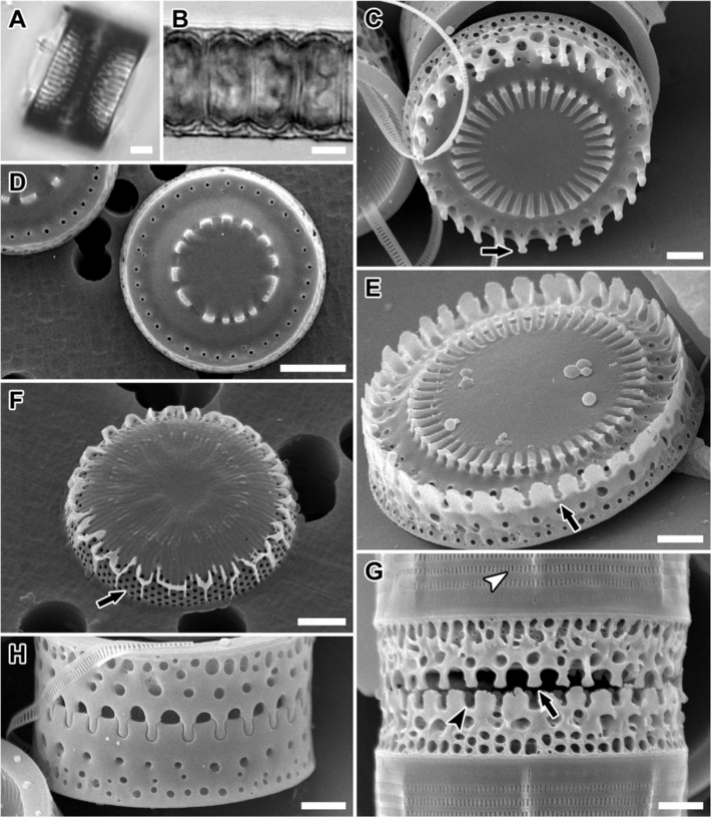
**LM and SEM images of** ***Paralia marina*** **. (A,B)** CCAP 1059/2. Light micrographs. **(A)** Interlocked sibling valves showing narrow fenestrae. **(B)** Chain of live cells showing the outline of the cell lumen. **(C-H)** Scanning electron micrographs. Epitype specimens, B40 0040792. **(C-F)** Valve face view. **(C)** Uru6. Smooth intercalary valve in relief showing long, slender and capitate marginal linking spines (arrow). **(D,E)** Intaglio valves (square-shouldered, short and blunt spines [12]). **(D)** Par1. Separation valve with smooth valve face and unadorned margin. **(E)** Intercalary valve showing marginal ridge formed by blunt, short spines with notches (arrow) between them which interlock with end of long, slender marginal spines. **(F)** Uru1. Incompletely silicified separation valve with rows of pores in a decussate pattern on the valve mantle (arrow). **(G,H)** Girdle view of sibling intercalary valves. **(G)** Helgo3. Nearly interlocked valves with long, slender capitate marginal linking spines (arrow), narrow fenestrae exposed, marginal square-shouldered, blunt spines with notches (black arrowhead) and simple slits in copulae (white arrowhead). **(H)** Uru6. Completely silicified interlocked sibling valves with fenestrae obscured. Scale bars = 5 μm **(B,D)**, 3 μm **(A)**, 2 μm **(C,E-H)**.

**Figure 4:**
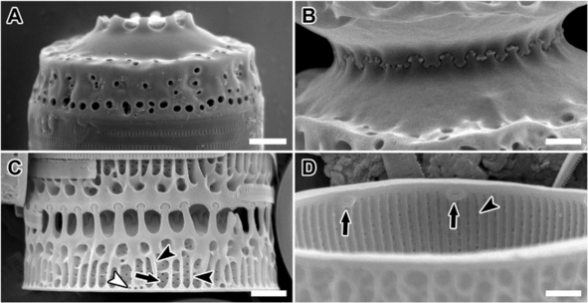
**SEM images of** ***Paralia marina*** **.** Epitype specimens, B40 0040792. **(A-C)** Mantle view. **(A)** Uru1. Profile of smooth and spineless separation valve with obscured fenestrae. **(B)** Helgo1. Interlocking noduliform protrusions of sibling separation valves. **(C)** Helgo3. Weakly silicified intercalary valves showing areolae perforating basal silica layer (arrow), siliceous outcroppings (black arrowheads) and external pores of rimoportulae (white arrowhead) which are usually more spaced apart than on this specimen; fenestrae unobscured. **(D)** Helgo1. Internal valve view with rimoportulae (arrow) and striae pores (arrowhead). Scale bars = 2 μm **(A,C)**, 1 μm **(B,D)**.

**Figure 5:**
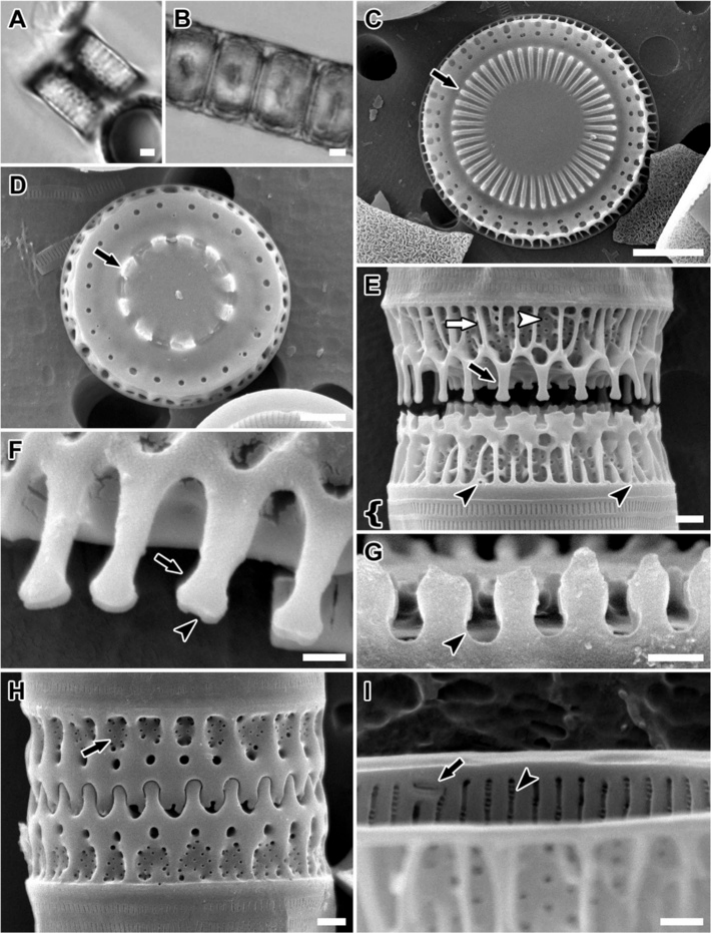
**LM and SEM images of** ***Paralia guyana*** **‘smooth’ valves. (A,B)** SV7. Light micrographs. **(A)** Holotype specimen of *P. guyana*, CANA 107802. Smooth valve face sibling separation valves. **(B)** A chain of live cells. **(C-I)** Scanning electron micrographs. Isotype specimens of *P. guyana* B40 0040793. **(C,D)** Valve face of intercalary (C; EC112-3) and separation (D; EC106-1) valves with arrows marking central baculiform elevations and noduliform central protrusions, respectively. **(E)** EC105-2. Mantle view of relatively weakly silicified and slightly separated sibling intercalary valves with unobscured fenestrae. Black arrow indicates long, capitate marginal linking spines. Black arrowheads point to external pores of rimoportulae. Siliceous outcropping (white arrow) and pore (white arrowhead) in basal silica layer are also evident. Parenthesis indicates cingulum. **(F)** YH1. Marginal linking spine (arrow) with slit at its apex (arrowhead). **(G)** EC109-2. Notches (arrow) between the short, blunt, squared-shoulder spines in relief intercalary valve. **(H)** Van4B3. Mantle view of interlocked sibling intercalary valves with arrow denoting shallow depressions in the siliceous covering of fenestrae. **(I)** GA3. Internal view of valve showing slit of rimoportulae (arrow) and striae pores (arrowhead). Scale bars = 2 μm **(A-D)**, 1 μm **(E,G,H)**, 0.5 μm **(F,I)**.

**Figure 6:**
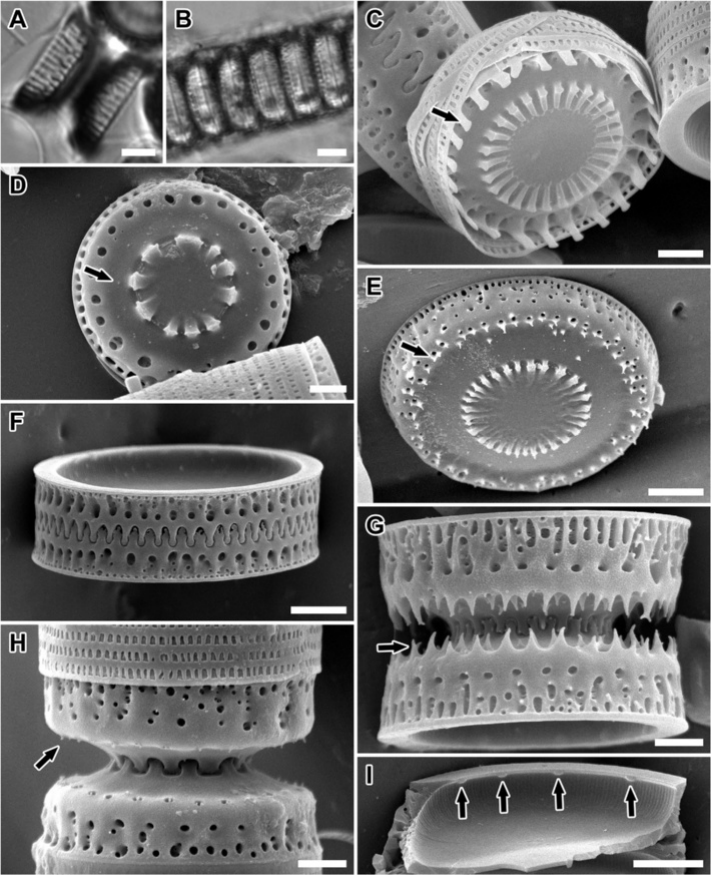
**LM and SEM images of** ***Paralia guyana*** **‘caisn’ valves. (A,B)** VanA4. Light micrographs. **(A)** Sibling separation valves. **(B)** A chain of live cells. **(C-I)** Scanning electron micrographs. Isotype specimens of *P. guyana* B40 0040794. **(C-E)** Valve face. **(C)** Van2B2. Intercalary valve with slightly capitate, long marginal linking spines (arrow) and copulae. **(D)** Van2B2. Nearly smooth separation valve with a few minute prickles (arrow). **(E)** Van4A2. Separation valve showing small, abundant prickles (arrow). **(F)** Van4A2. Interlocked sibling intercalary valves with obscured fenestrae. **(G,H)**. Interlocked sibling separation valves showing variation in size of prickles on the valve face margin. **(G)** West11B4. Well developed prickles (arrow); most fenestrae of topmost valve unobscured. **(H)** Van2B2. Minute prickles (arrow); obscured fenestrae. **(I)** Van4A2. Internal view of valve with irregularly spaced rimoportulae (arrows). Scale bars = 5 μm **(A,B,E,F,I)**, 2 μm **(C,D,G,H)**.

**Figure 7:**
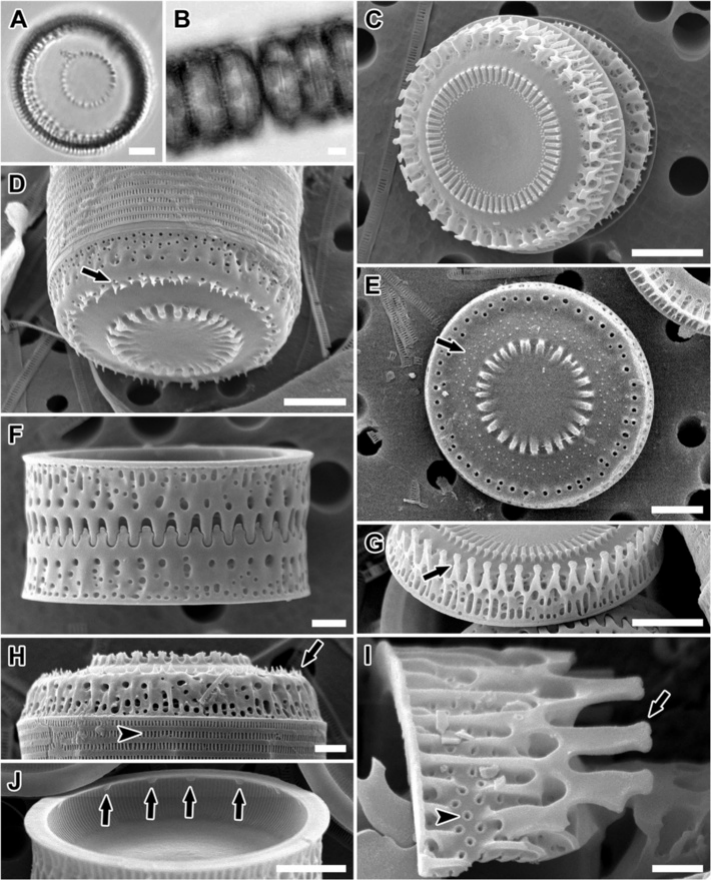
**LM and SEM images of** ***Paralia guyana*** **‘capebreton’ valves. (A,B)** GP1. Light micrographs. **(A)** Valve face view of separation valve. **(B)** A chain of live cells. **(C-J)** Scanning electron micrographs. Isotype specimens of *P. guyana* B40 0040795. **(C-E)** Valve face. **(C)** GP1. Intercalary valve. **(D)** GP6. Separation valve with marginal prickles (arrow). **(E)** GP2. Separation valve with minute prickles throughout valve face (arrow). **(F)** GP1. Interlocked sibling intercalary valves; fenestrae covered. **(G)** GP6. Tilted valve face view of intercalary valve with capitate marginal spines (arrow); fenestrae uncovered. **(H)** GP6. Mantle view of separation valve with prickles (arrow) and slits in copulae (arrowhead). **(I)** GP4. Portion of mantle of incompletely silicified intercalary valve with marginal spines (arrow), pores of basal silica layer (arrowhead) and exposed fenestrae. **(J)** GP5. Internal view of valve showing positions of rimoportulae (arrows). Scale bars = 5 μm **(A-E,G,J)**, 2 μm **(F,H)**, 1 μm **(I)**.

**Figure 8:**
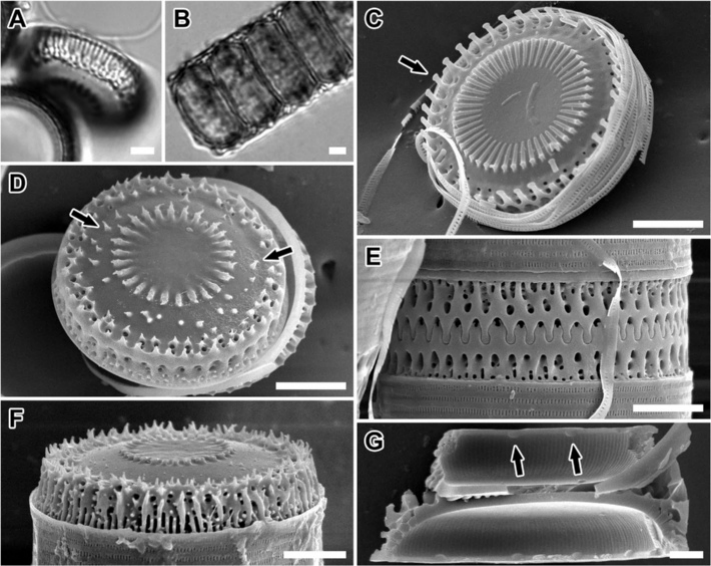
**LM and SEM images of** ***Paralia guyana*** **‘servidei’ valves. (A,B)** Van3C1. Light micrographs. **(A)** Tilted separation valve showing valve face and mantle. Note that even the characteristically pronounced prickles in this genodeme are not detectable on LM image; fenestrae notable. **(B)** A chain of live cells. **(C-G)** Scanning electron micrographs. Isotype specimens of *P. guyana* B40 0040796. **(C,D)** Van3C1. Valve face view. **(C)** Intercalary valve face with capitate, slender marginal spines (arrow). **(D)** Separation valve with pronounced prickles (arrows). **(E)** West1C2. Interlocked sibling intercalary valves and cingulae; upper valve with some fenestrae unobscured. **(F)** West1C2. Mantle view of relief separation valve showing prickles on the mantle. **(G)** West1C2. Internal view of valve with rimoportulae (arrows) and striae. Scale bars = 5 μm **(A-F)**, 2 μm **(G)**.

In total, 76 clonal isolates and 18 chains of *Paralia* (approximately 2000 valves altogether) were investigated from natural sediment and plankton samples worldwide and from ship ballast sediments and waters arriving at Atlantic Canadian ports (TAVs; Table 1). Consequently, two species were recovered; a previously described, but broadly delineated entity, *Paralia marina*, lectotypified here, and a species new to science, *P. guyana*, represented by four genodemes. These taxa were all morphologically semi-cryptic or cryptic (Figures 1, 2, 3, 4, 5, 6, 7, and 8) and were most reliably delineated based on DNA sequence data analyses.

**Table 1:** **Identities, sources and isolation dates of** ***Paralia*** **clones**

**Clone identifier**	**Site**	**Origin**	**Isolated**	**GenBank accession numbers**		
				**ITS**	**18S**	***rbc*** **L**
***P*** **.** ***marina***						
AC554	Baie des Veys, FR	49°23′N, 001°07′W	2002	KP150159	KP150015	KP150087
CAWB38	Croisilles Harbour, NZ	41°03′S, 173°39′E	01/01/98	KP150160	KP150016	KP150088
CCAP 1059/1^1^	Lynn of Lorn, UK	56°30′N, 005°28′W	01/12/02	KP150150	KP150008	KP150080
CCAP1059/2^1^	Loch Creran, UK	56°32′N, 005°20′W	2005	KP150152	KP150010	KP150082
Chet1	Cheticamp, CA	46°37′N, 061°00′W	13/08/09	KP150158	KP150014	KP150086
Helgo1^1,2^	Helgoland, DE	54°10′N, 007°53′E	20/02/07	KP150153	KP150011	KP150083
Helgo2^1,2^	”	”	06/03/07	KP150155	KP150012	KP150084
Helgo3^1,2^	”	”	03/07/07	KP150149	KP150007	KP150079
Helgo4^1,2^	”	”	06/12/07	KP150147	KP150005	KP150077
Par1^1,3^	Trieste, IT	45°42′N, 013°42′E	04/28/06	KP150143	N/A	KP150074
Par2^1,3^	”	”	04/28/06	KP150144	N/A	KP150075
***TAV1***	Multiple	Multiple	Multiple	KP150156	N/A	N/A
***TAV2***	”	”	”	KP150146	N/A	N/A
***TAV3***	”	”	”	KP150154	N/A	N/A
Uru1	Montevideo, UY	34°57′S, 056°09′W	31/07/09	KP150145	KP150004	KP150076
Uru2	”	”	31/07/09	KP150148	KP150006	KP150078
Uru3	”	”	31/07/09	KP150157	KP150013	KP150085
Uru6	”	”	31/07/09	KP150151	KP150009	KP150081
***P*** **.** ***guyana*** **‘smooth’ genodeme**						
**EC102-5**	Sydney, CA	46°08′N, 060°11′W	15/07/09	N/A	KP149985	KP150057
**EC105-2**	Portland, US	43°39′N, 070°15′W	03/08/09	KP150127	KP149986	KP150058
**EC106-1**	Boston, US	42°21′N, 071°03′W	07/08/09	N/A	KP149964	KP150036
**EC107-3**	”	”	07/08/09	KP150113	KP149972	KP150044
**EC108-1**	Belledune, CA	47°51′N, 065°51′W	07/08/09	KP150129	KP149988	KP150060
**EC109-2**	Botwood, CA	49°08′N, 055°21′W	07/08/09	KP150108	KP149966	KP150038
**EC110-2**	Stephenville, CA	48°33′N, 058°33′W	07/08/09	KP150124	KP149983	KP150055
**EC111-1**	Portland, US	43°39′N, 070°15′W	11/08/09	KP150115	KP149974	KP150046
**EC112-3**	Boston, US	42°21′N, 071°03′W	11/08/09	KP150122	KP149981	KP150053
**EC113-3**	Charlottetown, CA	46°14′N, 063°07′W	11/08/09	KP150111	KP149970	KP150042
**EC114-2**	St. John’s, CA	47°33′N, 052°42′W	11/08/09	KP150132	KP149991	KP150063
**EC115-1**	Boston, US	42°21′N, 071°03′W	13/08/09	KP150123	KP149982	KP150054
**EC116-4**	Bucksport, US	44°34′N, 068°47′W	13/08/09	KP150136	KP149995	KP150067
**EC117-1**	Corner Brook, CA	48°57′N, 057°57′W	27/08/09	KP150105	KP149962	KP150034
**EC118-1**	Sydney, CA	46°08′N, 060°11′W	27/08/09	KP150116	KP149975	KP150047
BH2	Bar Harbour, US	44°23′N, 068°12′W	12/08/09	KP150106	KP149963	KP150035
CB5	Chaleur Bay, CA	47°59′N, 066°40′W	03/07/09	KP150139	KP149998	KP150070
CT2	Cape Tormentine, CA	46°07′N, 063°47′W	03/07/09	KP150138	KP149997	KP150069
GA3	Grande Anse, CA	49°04′N, 068°24′W	06/06/09	KP150128	KP149987	KP150059
IngoB3	Ingonish, CA	46°42′N, 060°22′W	05/10/09	KP150134	KP149993	KP150065
Ingram	Ingramsport, CA	44°39′N, 063°58′W	25/10/09	KP150119	KP149978	KP150050
Mait1	Maitland Beach, CA	43°59′N, 066°09′W	08/09/09	KP150135	KP149994	KP150066
MB2	Maces Bay, CA	45°05′N, 066°28′W	14/08/09	KP150120	KP149979	KP150051
Min2	Minudie, CA	45°46′N, 064°21′W	03/07/09	KP150130	KP149989	KP150061
Moose3	Moose Cove, CA	45°37′N, 064°31′W	07/07/09	N/A	KP149969	KP150041
MP1	Mary’s Point, CA	45°41′N, 064°34′W	07/07/09	KP150133	KP149992	KP150064
NMB1	Noel, CA	45°17′N, 063°44′W	08/09/09	KP150118	KP149977	KP150049
PM3	Pretty Marsh, ME	44°20′N, 068°23′W	12/08/09	KP150110	KP149968	KP150040
Que 53-1	Baie aux Outardes, CA	49°04′N, 068°24′W	10/10/09	KP150112	KP149971	KP150043
SV7	Shediac Valley, CA	47°20′N, 064°25′W	03/03/09	KP150131	KP149990	KP150062
SV8	”	”	03/03/09	KP150137	KP149996	KP150068
SV9	”	”	03/03/09	KP150117	KP149976	KP150048
SV12	”	”	03/03/09	KP150126	N/A	N/A
SV14	”	”	03/03/09	KP150142	KP150001	KP150073
Van2A1	Ucluelet Peninsula, CA	48°55′N, 125°32′W	19/05/10	KP150140	KP149999	KP150071
Van4B3	Tofino, CA	49°09′N, 125°53′W	19/05/10	KP150114	KP149973	KP150045
Van5A1	Tsawwassen, CA	49°01′N, 123°06′W	19/05/10	KP150109	KP149967	KP150039
Van5A2	”	”	19/05/10	KP150141	KP150000	KP150072
W5	Windsor, CA	44°59′N, 068°08′W	28/05/09	KP150121	KP149980	KP150052
YCB4	North Sydney, CA	46°12′N, 060°15′W	03/10/09	KP150107	KP149965	KP150037
YH1	Yarmouth, CA	43°50′N, 066°06′W	08/09/09	KP150125	KP149984	KP150056
***P*** **.** ***guyana*** **‘caisn’ genodeme**						
VanA4	Nanaimo, CA	49°12′N, 123°57′W	31/01/10	KP150089	KP149947	KP150018
Van2B2	Ucluelet Peninsula, CA	48°55′N, 125°32′W	19/05/10	KP150091	KP149949	KP150020
Van4A2	Tofino, CA	49°09′N, 125°53′W	19/05/10	KP150094	KP149952	KP150023
Van4C1	”	”	19/05/10	KP150093	KP149951	KP150022
West11B4	Marshall, US	38°09′N, 122°53′W	27/05/10	KP150095	KP149953	KP150024
West11C2	”	”	27/05/10	KP150092	KP149950	KP150021
West11C3	”	”	27/05/10	KP150090	KP149948	KP150019
***P*** **.** ***guyana*** **‘capebreton’ genodeme**						
GP1	Grove’s Point, CA	46°13′N, 060°20′W	12/08/09	KP150096	KP149954	KP150025
GP2	”	”	12/08/09	KP150098	KP149956	KP150027
GP3	”	”	12/08/09	KP150099	KP149957	KP150028
GP4	”	”	12/08/09	KP150097	KP149955	KP150026
GP5	”	”	12/08/09	KP150101	KP149959	KP150030
GP6	”	”	12/08/09	KP150100	KP149958	KP150029
***P*** **.** ***guyana*** **‘servidei’ genodeme**						
Van3C1	Ucluelet Inlet, CA	48°56′N, 125°33′W	19/05/10	KP150102	N/A	KP150031
West1C2	Aberdeen, US	46°58′N, 123°48′W	20/05/10	KP150103	KP149960	KP150032
West3B3	Waldport, US	44°25′N, 124°04′W	20/05/10	KP150104	KP149961	KP150033
***P*** **.** ***allisonii***						
Jamaica2	Dunns River Falls, JM	18°24′N, 077°08′W	13/04/10	JN201575	JN201583	JN201591
PanamaA3	”	”	13/04/10	JN201577	JN201585	JN201593
***P*** **.** ***crawfordii***						
MexSmDia1	Baja California Sur, MX	26°12′N, 111°18′W	15/09/09	JN201579	JN201587	JN201595
***P*** **.** ***fenestrata***						
MexLgDia	”	”	15/09/09	N/A	N/A	KP150017
***Stephanopyxis palmeriana***						
CCMP0814	Gulf of Mexico, US	28°37′N, 89°45′W	02/07/80	KP193457	AY485527	KP253080

GenBank accession numbers for ITS, 18S, and *rbc*L sequences. ^1^As *P. sulcata*; ^2^Cultures provided by K. Gebühr from the Alfred Wegener Institute; ^3^Cultures provided by A. Beran from OGS/BIO Trieste. Bold clone identifiers indicate specimens from the ship ballast, italicised are ballast waters, non-italicised are sediments; N/A = Sequence data not available.

***Paralia marina*** (W. Smith) Heiberg (Figures 2A-H; 3A-H; 4A-D; Table 2).

**Table 2:** **Summary of metric characters of** ***Paralia*** **species in this study**

	***P*** **.** ***marina***	***P*** **.** ***guyana***			
**Genodeme**		**‘smooth’**	**‘caisn’**	**‘capebreton’**	**‘servidei’**
# of clones examined	15	41	7	6	3
Character					
Valve diameter (μm)	12.2 (3.4);	11.5 (2.9);	15.3 (5.2);	18.6 (4.2);	19.9 (2.5);
	5.4-20.2; 199	6.9-22.9; 571	9.0-26.9; 147	9.2-23.8; 109	13.7-24.1; 47
Pervalvar axis (μm)	7.6 (1.1);	7.7 (1.1);	15.3 (5.2);	10.4 (4.4);	7.6 (0.99);
	5.5-9.7; 31	4.9-11.3; 132	9.0-26.9; 14	6.4-10.3; 17	6.5-8.5; 5
**Valve face**					
Internal linking spines in 10 μm	5.9 (2.5);	6.4 (3.1);	6.0 (2.9);	7.1 (2.9);	7.0 (3.3);
	2.3-11.5; 116	1.2-11.9; 312	2.8-11.7; 83	3.2-12.0; 68	2.2-13.8; 32
Separation valve prickles present	No	No	Mostly	Mostly	Mostly
**Valve mantle external**					
Marginal linking spines in 10 μm	7.7 (0.87);	8.2 (0.92)	7.8 (0.78)	7.7 (0.81)	7.6 (0.67)
	5.9-10.0; 42	5.7-10.4; 197	6.5-10.2; 23	6.0-9.9; 38	6.4-8.8; 14
Fenestrae in 10 μm	10.5 (3.4);	8.0 (0.81);	9.9 (3.9);	7.6 (0.6);	7.9 (1.2);
	6.0-15.4; 30	5.6-10.3; 191	5.9-18.1; 17	6.4-8.6; 42	6.4-8.9; 4
Cingulum slits in 10 μm	51.6 (6.4);	53.8 (6.7);	41.2 (6.8);	44.7 (4.4);	42.6 (7.8);
	38.5-68.7; 108	33.5-76.1; 335	33.1-61.0; 162	36.8-61.0; 103	33.7-66.3; 16
**Valve mantle internal**					
Internal striae in 10 μm	37.4 (4.7);	38.6 (3.3);	38.0 (2.7);	37.7 (2.1);	38.9 (3.3);
	31.1-60.0; 54	32.8-64.3; 177	30.3-43.0; 36	33.7-42.7; 40	34.3-45.6; 14
Internal striae pores in 1 μm	3.0 (0.34);	2.8 (0.33);	3.5 (0.41);	2.8 (0.30);	3.05 (0.38);
	2.7-3.6; 5	2.3-4.1; 71	3.0-4.0; 8	2.4-3.3; 14	2.8-3.5; 3
Rimoportulae in 10 μm	1.9 (0.53);	2.1 (0.48);	2.5 (0.48);	2.5 (0.47);	2.4 (0.28);
	1.1-2.5; 26	0.4-3.6; 87	1.5-3.8; 27	1.5-3.5; 25	1.9-2.9; 11

Values given for mean with standard deviation in parentheses, followed by range and number of measurements.

*Lectotype* (designated here). Drawing of sibling valves from Smith ([17], pl. 53, figure 338); labelled as ‘7’ on Figure 2A. The valve noted above most closely represents those found on Smith’s preparation currently at the British Museum BM1021; no specimen was marked on this preparation.

*Lectotype locality*. Kinsale Harbour, Ireland (indicated on preparation BM1021 as such). Smith’s [17] diagnosis reads ‘Filaments direct; cell-cavity sub-spherical; valve with large irregular cellules, and a deep submedian sulcus or depression; junction-surfaces striated; striae radiating. Breadth of filament •0006“ to •0018”. v.v.’ The •0006“ to •0018” v.v. given in the original description corresponds to 15.2-45.7 μm valve diameter in units used in modern diatom taxonomy.

*Epitype* (designated here). SEM preparation B40 0040792 from strain 1059/2 (labelled as *Paralia sulcata*) from the Culture Collection of Algae and Protozoa (CCAP), deposited at The Botanic Gardens and Botanical Museum (BGBM) in Berlin-Dahlem, Berlin, Germany. The entire ITS region and sequence fragments of the *rbc*L and 18S rRNA genes and voucher specimens are deposited in GenBank via the Barcoding of Life Data (BOLD) System (accession numbers in Table 1).

*Epitype locality*: Isolated from Loch Creran, Argyll, Scotland (56°32′N, 05°20′W) as given at CCAP for CCAP 1059/2.

*Rationale for lecto- and epitypification*: As was common practice at the time, Smith did not establish the holotype for *Orthosira marina* (transferred to *Paralia* by Heiberg [18]). The Botanical Code [45], and Jahn & Kusber [46] in the context of diatoms, state that: ‘a specimen from its original material such as pictures, isotypes (duplicates) or syntypes’ may be used as a lectotype when no holotype has been designated (as is the case here), providing the material was in the hands of the describer at the time of description. Crawford [15] has noted that ‘Since Heiberg stated definitively that the type of *Paralia* is *Orthosira marina* W. Smith the British Museum slide, number 1021, must be regarded as a lectotype of *Paralia*’. Because Smith’s BM1021 preparation contains no marked specimen, one of Smith’s [17] drawings (pl. 53, figure 338, marked as 7 here; cells sizes not given) was designated as the lectotype for *P. marina*. However, figure 338 [17] seems to show two morphotypes. This might have contributed to ambiguities surrounding the status of this species and its relationship to the earlier described *P. sulcata*. Indeed, drawings labelled 5, 6 and 8–10 here (Figure 2A) have the characteristic wide, open fenestrae of Miocene *P. sulcata s.s*. and extant *P. fenestrata* (compare to figure 33 of [10]). The drawings labelled 1–4 and 7 here (Figure 2A), on the other hand, have narrow fenestrae that are more characteristic of those seen in the *Paralia* valves on preparation BM1021 (Figure 2B-I, figures seven to nine in [15]); no unmounted material examined by Smith was available to the authors.

Although two contemporary species, *P. marina* and *P. guyana* (described below), share many characters of the intercalary frustule morphology discernible in LM and are comparable to LM images of Smith’s BM1021 preparation (Figure 2B-I), the epitype of *P. marina* was chosen from the clone established from Loch Creran, Scotland (CCAP 1059/2). The site, approximately 500 km away, was the one nearest to where the sample for preparation BM1021 was obtained, Kinsale Harbour, Ireland; although there is no guarantee that the *Paralia* sampled by W. Smith in the mid-1800s still exists at this site.

*Emended description*: Valves circular and heavily-silicified. Separation valve face (shown to carry the best discriminating characters in this and other studies) is devoid of prickles or marginal spines. On intercalary valves, long, slender, capitate marginal linking spines interdigitate with notches between the square-shouldered, short and blunt spines (*sensu* [12]) in sibling valves to form filamentous colonies. Fenestrae, two areolae wide, are bordered by siliceous outgrowths and occur on the valve mantle. They are obscured by a siliceous covering in fully silicified valves when observed in SEM. Internally, the mantle rim is smooth. Just below the overhanging mantle edge are irregularly spaced rimoportulae which, when present, replace a few pore areolae in striae that otherwise run the length of the mantle. Nuclear rDNA ITS2 secondary structure of this diatom has five helices, a C:U mismatch in Helix II and a UGGU super-conserved motif on the 5′ side of Helix III (compare to [6], figure 45, ellipse).

*Morphometrics of valves on preparation BM1021*: Overall, 97 valves were examined and the following characters were measured: valve diameter (n = 97; $\overline{x}$ = 16.6 μm; range = 10.8-33.2 μm), the number of fenestrae (n = 19; $\overline{x}$ = 15.6 in 10 μm; range = 12.4-17.9 in 10 μm), and the marginal linking spines (n = 12; $\overline{x}$ = 8.1 in 10 μm; range = 6.6-9.6 in 10 μm).

*Detailed description of frustule morphology based on SEM images of our clones*: In the 15 cultures examined, straight chains were formed of interlocking cells (Figure 3A); each cell contained 4–6 discoid chloroplasts (Figure 3B). Chains could often reach > 100 cells, but most were < 30 cells. Frustules were cylindrical and strongly silicified, 5.4-20.2 μm in diameter and 5.5-9.7 μm in pervalvar axis. Two types of valves were found, intercalary (Figures 3C, E, G, and H; 4C) and separation (Figures 3D; 4A and B), each with two forms, relief (long, slender marginal spines, Figures 3E; 4A) and intaglio (short and blunt marginal spines, Figure 3C and D). Capitate marginal linking spines (5.9-10.0 in 10 μm; Figure 3C and G, arrow), occurred only along the face margin of intercalary valves and fit into notches (Figure 3E [arrow] and G [black arrowhead]) between the marginal, short and blunt, square-shouldered spines of a sibling valve. An inner ring of baculiform elevations or internal linking spines of varied size, 2.3-11.5 in 10 μm, tapered in height towards the unadorned valve centre. The “internal” linking spines are so named because they are internal relative to the marginal linking spines. The marginal and internal linking spines of sibling valves interlocked to keep cells in colonies (Figure 4C), albeit with a different “holding” strength. Marginal linking spines are absent on separation valves. Normally, two concentric rings of larger and smaller pores were present at the valve face margin of intercalary valves (Figure 3C and E).

Separation valves had smooth valve faces. They carried only a pericentral ring of internal spines in the form of noduliform protrusions (Figure 3D) connecting sibling separation valves (Figure 4B). Generally, only one ring of regular, medium-size pores was present on these valves (Figure 3D). The valve face and the mantle met at an approximately right angle, forming a relatively flat, cylindrical girdle outline of the frustule (e.g., Figure 4A).

All valves’ basal silica layer was perforated by small, regularly-organised poroid areolae in a decussate pattern of pores (Figure 3F, arrow), still visible on the mantle in fully formed valves of some specimens (Figure 4C, arrow); 2.7-3.6 in 1 μm (Figure 4D, arrowhead). Externally, the pairs of mantle striae were separated by siliceous outcroppings (Figure 4C, black arrowheads) superimposed over the basal silica layer. An interspaced pair of striae and siliceous outcroppings formed narrow fenestrae (6.0-15.4 in 10 μm). Mantle fenestration was most pronounced in incompletely silicified valves (compare Figures 3G and 4C with 3H) and when observed in SEM. In fully silicified valves, fenestrae were cached with a siliceous cover dotted with simple pores (Figure 3H). Even though fenestrae were obscured in SEM, they were nonetheless generally resolvable in LM (Figure 3A). Cingulae were composed of 8–9 copulae and each copula carried regularly-spaced, centrally-located slits (Figure 3G, white arrowhead), 38.5-68.7 in 10 μm.

Internally, the mantle rim was smooth (Figure 4D). Irregularly-spaced, simple and small rimoportulae, 1.1-2.5 in 10 μm, were positioned just below the mantle overhanging edge (Figure 4D, arrows). Minute external openings of the rimoportulae were observed in many valves (Figure 4C, white arrowhead). Internally, striae began below the mantle edge and ran perpendicular to it but few pore areolae were replaced by rimoportulae when they were present (31.1-60.0 in 10 μm). Striae varied in length and did not extend onto the face of the completely silicified valve. A summary of the clone metrics is presented in Table 2.

**Figure 9:**
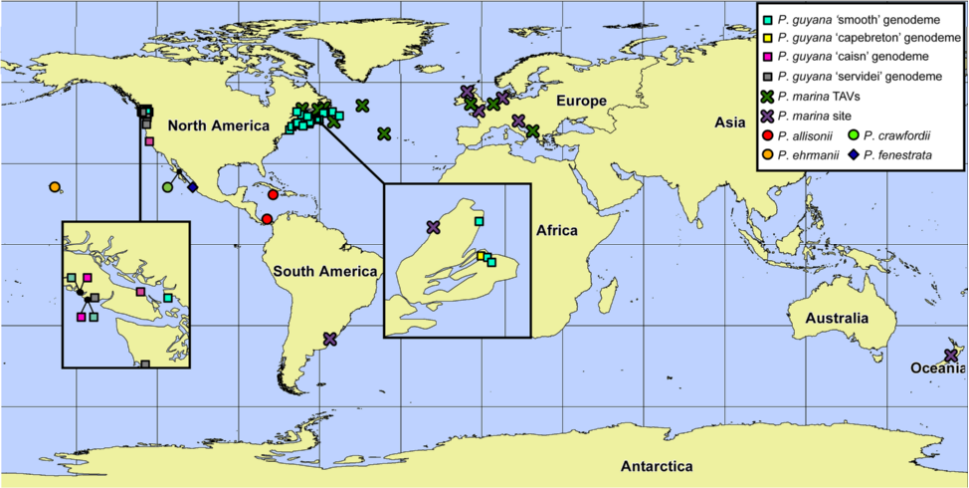
**Distribution of** ***Paralia*** **species recovered in this study and in MacGillivary and Kaczmarska [** 6 **,** 10 **].** Inserts show hotspots of species richness. Leftmost is Vancouver Island, Canada and rightmost is Cape Breton Island, Canada. Leader lines to inserts emanate from original location on world map. *P. marina*, specimens collected from natural waters are denoted as ‘site’ while those collected on trans-Atlantic voyages are denoted as ‘TAVs’.

*Distribution*: *Paralia marina* was the most widely distributed species of the *Paralia* taxa examined in this study: five sites throughout Europe and one site in Uruguay, New Zealand and eastern Canada (Table 1, Figure 9). In addition, chains of *P. marina* was recovered throughout all three TAVs (96% of total cells encountered) with an average propagule size of 192 cells l^−1^ on the final day of each TAV.

*Comparison to morphology of other species*: *Paralia marina* is morphologically indistinguishable from the ‘smooth’ extant *P. guyana* genodeme (described below), from Miocene *P. obscura* MacGillivary [10] and from extant natural specimens named *P. sulcata* (illustrated in figures eleven to twenty-five in [12]). Unfortunately, genetic data is not available for the latter two entities and therefore their relationship to *P. marina* cannot be resolved. *Paralia marina* differs from the members of the *Paralia longispina* species complex by the absence of marginal spines on separation valves. Also, fenestrae are more regularly elliptical-shaped in *P. longispina*-like species than in *P. marina.* Fenestrae shape and size again differ between *P. marina* and *P. fenestrata* and recently lectotypified *P. sulcata* [10] as the latter two have U-shaped fenestrae that are exposed (not covered by a siliceous overlayer) even in completely silicified valves. Unfortunately, fenestra morphology on valves of *P. sulcata s.s*. with similar cell sizes as *P. marina* examined here is unknown at this time, but in *P. fenestrata* even very small valves demonstrate open, large fenestra (Kaczmarska, unpublished results), clearly different from those of *P. marina*. Unlike *P. elliptica*, which has an elliptical to kidney-shaped valve face, *P. marina* has a circular outline. Marginal linking spines of intercalary valves are deeply capitate in *P. capitata* in contrast to subtle capitate spines in *P. marina*.

Molecularly, *P. marina* is quite divergent from *P. guyana* (see below), *P. allisonii* and *P. crawfordii* [6] in the examined sequences of 18S (d = 0.02 [representing two base pair (bp) differences per 100 bp sequence segment], d = 0.02, d = 0.03, respectively), ITS (d ≥ 0.34 [depending on the genodeme], d = 0.35, d = 0.37, respectively) and *rbc*L (d ≥ 0.05 [depending on the genodeme], d = 0.05, d = 0.06, respectively). There are also structural differences in ITS2 secondary structure models between *P. marina* and the other three species; *P. marina* has five helices whereas the other species have four. In addition, even the superconserved motif on the 5′ side of Helix III differs between *P. marina* (UGGU), *P. allisonii* and *P. crawfordii* (AGGU, [6]) and *P. guyana* (AGGA). Furthermore, Compensatory Base Changes (CBCs) are present between *P. marina* vs. *P. allisonii* and *P. marina* vs. *P. guyana*; basal portions of each in Helix I and Helix IV and near the terminal loop in Helix II, which are the few areas of relative conservation.

***Paralia guyana*** MacGillivary **sp. nov.** (Figures 5, 6, 7, 8; Table 2).

*Holotype*: Material of culture “SV7” cleaned and Hyrax embedded slide (CANA 107802), National Herbarium of Canada, Phycology Section, Canadian Museum of Nature, Ottawa, Canada. Holotype specimen illustrated in Figure 5A. The entire ITS region and sequence fragments of the *rbc*L and 18S rRNA genes and voucher specimens were deposited in GenBank via the BOLD System (accession numbers in Table 1).

*Type locality*: Phytoplankton sample from Shediac Valley, Gulf of St. Lawrence, Canada (47°20′N, 64°25′W).

*Isotype*: SEM preparation B40 0040793 (Figure 5, C-I; Figure 6, C-I; Figure 7, C-J; Figure 8, C-G) *ex cultura*, deposited at the BGBM.

*Etymology*: Species dedicated to the first author’s maternal grandparents (surname Guy) on the occasion of their 55th wedding anniversary.

*Diagnosis*: Valves circular, forming straight chains. Separation valve face free of marginal spines, but may carry prickles. In SEM preparations, fenestrae obscured with a siliceous covering in intact specimens. Secondary structure of the nuclear rDNA ITS2 transcript has four helices, a combined U:C and C:C mismatch in Helix II and AGGA super-conserved motif on the 5′ side of Helix III.

*Description of frustule morphology*: Fifty-seven clones were examined for this species. Both intercalary and separation valves were morphologically indistinguishable from that of *P. marina* (above), both internally and externally in all 10 metric characters examined (Table 2). The only non-metric morphological difference found was that some separation valves of this species carried prickles [Figure 6D, E, G and Figure 7D, E, H and Figure 8D, arrow(s)] of various size, in addition to those valves that had smooth valve faces (Figure 5D). When present, prickles were most commonly located between the valve face margin and the pericentral ring of noduliform protrusions or on the mantle (i.e., Figure 8F).

*Distribution*: Recovered from 37 sites on the Atlantic and Pacific coasts of North America (Table 1, Figure 9).

*Comparison to other species*: This species is morphologically semi-cryptic or cryptic (depending on which of the four genodemes are used in the comparison, see below), yet molecularly it is strongly divergent from *P. marina* (see above and below). Comparison of the ITS2 secondary structures of all species for which they are available (*P. guyana* vs. *P. allisonii* and *P. guyana* vs. *P. crawfordii*) yields two and one CBC(s) in the basal part of Helix IV, respectively, among other differences (compare ITS2 secondary structures in [6], figure 45 ellipse). Within *P. guyana* there were four molecularly, and in one case morphologically, defined demes. They are described immediately below.

***Paralia guyana*** ‘smooth’ genodeme (Figure 5A-I).

Material: Grown in culture, the same as *P. guyana s.l*. (above).

Morphology: Aside from having a smooth separation valve face, this genodeme was morphologically cryptic with the other genodemes of *P. guyana*. It is also morphologically cryptic in both separation and intercalary valves with *P. marina*. See Table 2 for morphometric ranges.

Molecular signature: Compared to other genodemes of *P. guyana*, ‘smooth’ had an autapomorphy in helices I and II of ITS2: 5′-CUUUGUCUUGCGUUGGCCUGUGUCGCGGACC-3′ (31 bp).

Distribution: Found in 30 sites on the Atlantic and Pacific coasts of North America (Table 1, Figure 9).

***Paralia guyana*** ‘caisn’ genodeme (Figure 6A-I).

Material: Culture “VanA4” cleaned and Hyrax embedded slide (CANA 107803), National Herbarium of Canada, Phycology Section, Canadian Museum of Nature, Ottawa, Canada. SEM preparation B40 0040794 deposited at the BGBM.

Morphology: Had variable-sized prickles on the separation valve face which were only detectable in SEM. Morphologically cryptic with the ‘capebreton’ and ‘servidei’ genodemes, (see below and in Table 2).

Molecular signature: Autapomorphy in helices I and II of ITS2: 5′-CUGUGUCUUGAGUUGGCCUGUGUCGCGGAGC-3′ (31 bp). In combination, three transversions at sites 22, 30 and 49 of this sequence and transversions (site 371) and transitions (sites 439 and 475) in the *rbc*L fragment differentiated it from the other genodemes. The entire ITS region and sequence fragments of the *rbc*L and 18S rRNA genes and voucher specimens were deposited in GenBank via the BOLD System (accession numbers in Table 1).

Distribution: Found at four sites on the Pacific coast of North America (Table 1, Figure 9).

***Paralia guyana*** ‘capebreton’ genodeme (Figure 7A-J).

Material: Culture “GP1” cleaned and Hyrax embedded slide (CANA 107804), National Herbarium of Canada, Phycology Section, Canadian Museum of Nature, Ottawa, Canada. SEM preparation B40 0040795 deposited at the BGBM.

Morphology: Had variable-sized prickles on the separation valve face which were only detectable in SEM. Morphologically cryptic with the genodemes ‘caisn’ (above) and ‘servidei’ (below), and in Table 2.

Molecular signature: Autapomorphy in helices I and II of ITS2: 5′-CAUUGUCUUGCGUUGGCCUGUGUCGCGGAGC-3′ (31 bp). Combined, two transversions at sites 21 and 49 of this sequence differentiate it from other demes. The entire ITS region and sequence fragments of the *rbc*L and 18S rRNA genes and voucher specimens were deposited in GenBank via the BOLD System (accession numbers in Table 1).

Distribution: Found at one site in a salt-water lake in eastern Canada (Table 1, Figure 9).

***Paralia guyana*** ‘servidei’ genodeme (Figure 8A-G).

Material: Culture “Van3C1” cleaned and Hyrax embedded slide (CANA 107805), National Herbarium of Canada, Phycology Section, Canadian Museum of Nature, Ottawa, Canada. SEM preparation B40 0040796 deposited at the BGBM.

Morphology: Had stout, variable-sized prickles on the separation valve face which were only detectable in SEM when prickles were short. It is morphologically cryptic with genodemes ‘caisn’ and ‘capebreton’, (see above and in Table 2).

Molecular signature: Six hemi-compensatory base changes, one in Helix II, two in Helix III and three in Helix IV of the ITS2 transcript secondary structure differentiate it from other genodemes. The entire ITS region and sequence fragments of the *rbc*L and 18S rRNA genes and voucher specimens were deposited in GenBank via the BOLD System (accession numbers in Table 1).

Distribution: Found at three sites on the Pacific Northwest coast of North America (Table 1, Figure 9).

#### Morphometric analyses

**Figure 10:**
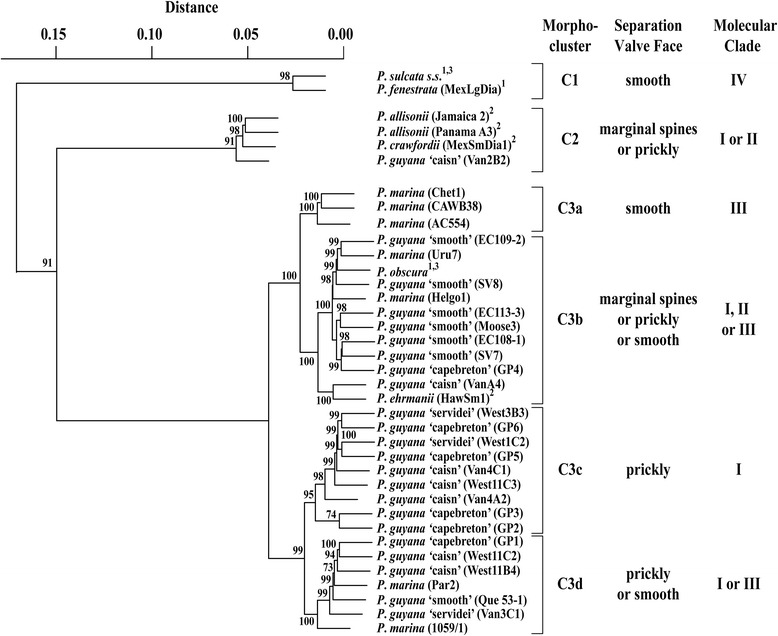
**Hierarchical clustering of** ***Paralia*** **species based on SEM morphometrics.** Representative clones were selected based on methods described in the text. The identity and taxonomic affiliation of each clone or sample (in parenthesis) are presented to the right of the cluster diagram. The assigned morpho-cluster and molecular clade (see below) and character state of separation valve face are indicated to the right. Multiscale bootstrap values (AU) are shown as p-values (multiplied by 100) generated from 10000 bootstrap samples; p ≥ 0.95 is considered statistically significant. Distance is equal to 1-correlation so that nodes closer to 0.00 are morphologically more similar. 1 = [10]; 2 = [6]; 3 = *P. sulcata s.s*., *P. ehrmanii* and *P. obscura* have not been genetically defined.

Eight species, two from this study and six from related species examined earlier [6,10], were analyzed metrically using SEM images using ten metric valve characters also used in recent taxonomic studies of this genus ([6,10,12]; Table 2). This is much more than a routine diatom metric analysis (e.g., valve length, width, striae and areolae) and demonstrated both the wide range of intraspecific morphological variation and the considerable overlap between these taxa. Hierarchical clustering of morphometrically continuous data (i.e., number of rimoportulae in 10 μm, Table 2) rendered approximately unbiased (AU) multiscale bootstrap values and ordered clones into three main clusters, namely, C1, C2 and C3 (Figure 10), represented by two, four and 31 clones respectively. The smallest cluster (p = 0.98), C1, consisted of Miocene *P. sulcata s.s.* and extant *P. fenestrata* which both had smooth valve faces on separation valves, but could be distinguished from all other taxa by open, wide, horseshoe-shaped fenestrae. The larger, second cluster, C2 (p = 0.91), included the *P. longispina*-like species, *P. allisonii* and *P. crawfordii*, both of which had triangular marginal spines on the face of separation valves, and one clone from the *P. guyana* ‘caisn’ genodeme which did not have marginal spines, but had prickles on the separation valve face (Figure 10). Cluster C3 contained four species and all genodemes of *P. guyana* and may be subdivided into four metrically defined sub-clusters containing a mixture of species and their genodemes. Each of the sub-clusters (C3a-d) had separation valves possessing various non-metric characters (i.e., presence or absence of prickles or spines on the separation valve face). Sub-cluster C3a contained only *P. marina* (p = 1.00). On the other hand, sub-cluster C3b (p = 1.00) grouped together four taxa with different separation valve face microarchitecture; smooth (Miocene *P. obscura*, extant *P. marina* and six clones of the extant ‘smooth’ genodeme of *P. guyana*), prickly (one clone each of the extant ‘caisn’ and ‘capebreton’ genodemes of *P. guyana*) and spiny (extant *P. ehrmanii*). The third sub-cluster, C3c (p = 0.95), contained the three prickly genodemes of *P. guyana* (three, four and two clones of the ‘caisn’, ‘capebreton’ and ‘servidei’ genodemes, respectively). Sub-cluster C3d grouped together seven clones representing the smooth-faced *P. marina* (two clones), the ‘smooth’ genodeme of *P. guyana* (one clone) and the three prickly separation valve-faced *P. guyana* genodemes (p = 1.00). Neither metric nor discrete characters measured and evaluated consistently segregated taxa from cluster C3 in agreement with their genetic signatures (see below).

### Sequence analysis

Amplicons from all three DNA regions, 18S, *rbc*L and ITS1, were recovered from all 76 clones (except for 18S from two clones of *Paralia marina* from Trieste, Italy; Table 1). For the 18 chains of *Paralia* from the TAVs, only the most divergent marker, the ITS region was amplified and analyzed. No intraspecific divergence among *P. marina* clones and no signs of heteroplasmy among *P. marina* and *P. guyana* demes were found for any of the three sequence regions. The interspecific divergence in the conserved region of 18S RNA gene fragment was 0–7 bp (0-2%). All *P. guyana* genodemes were 100% identical in the 18S segment but all were 2% divergent from *P. marina*. Interspecifically, there were 0–30 bp (0-6%) differences in the *rbc*L fragments, depending on the genodeme. The ‘capebreton’ and ‘servidei’ genodemes of *P. guyana* showed 100% identity while *P. marina* and the three prickly genodemes of *P. guyana* had the highest divergence (Additional file 1).

For the ITS region, the lowest sequence divergence occurred between the Pacific ‘caisn’ and Atlantic ‘capebreton’ genodemes of *P. guyana* (2%) whereas the highest occurred between the *P. guyana* ‘smooth’ genodeme and *P. marina* (36%). ITS1 sequence divergences were between 10–113 bp (2-34%) for the *P. guyana* ‘caisn’ and *P. guyana* ‘servidei’ genodemes (both from the Pacific coast) and the ‘smooth’ genodeme of *P. guyana* and *P. marina*, respectively. The ‘servidei’ and ‘caisn’ genodemes of *P. guyana* shared 100% identity in the 5.8S gene sequence, whereas the ‘capebreton’ genodeme of *P. guyana* and *P. marina* were the most diverse; 9 bp or 5%. The ITS2 marker was the most divergent sequence region examined. The *P. guyana* ‘smooth’ and ‘caisn’ genodemes were the most similar (4 bp or 1%) and *P. marina* and the *P. guyana* ‘servidei’ genodeme were the most diverse (138 bp or 58%). For the 5.8S + ITS2 barcode region *sensu* [47,48] the sequence divergences ranged from 1% between the ‘smooth’ and the ‘caisn’ genodemes of *P. guyana* to 25% between *P. marina* and the ‘capebreton’ and ‘caisn’ genodemes of *P. guyana*.

#### ITS2 secondary structures

**Figure 11:**
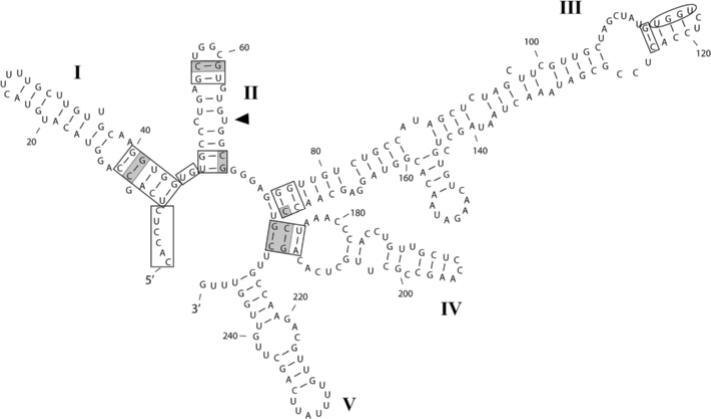
**ITS2 rRNA transcript secondary structure model of** ***Paralia marina*** **(strain Helgo3).** Sequence orientation (5′ to 3′) and helix numbering (I, II, III, IV and V) are specified. An arrowhead shows the pyrimidine-pyrimidine mismatch (CxU) on Helix II, the ultra-conserved UGGU motif on the 5′ side of Helix III is indicated by an ellipse and areas of relative conservation between *P. marina* and *P. guyana* are boxed. CBCs between *P. guyana* and *P. marina* are shaded gray across the entire base pair. HCBCs between *P. guyana* and *P. marina* are shaded only on the changed base of the base pair.

**Figure 12:**
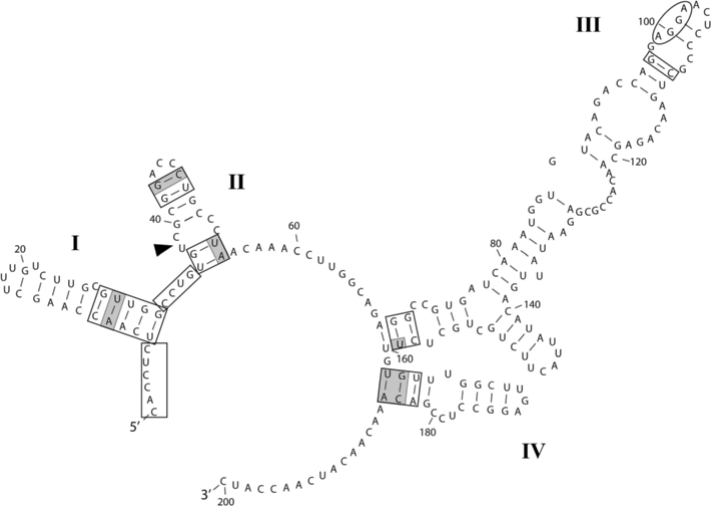
**ITS2 rRNA transcript secondary structure model of** ***Paralia guyana*** **(strain SV7).** Sequence orientation (5′ to 3′) and helix numbering (I, II, III and IV) are specified. An arrowhead shows the pyrimidine-pyrimidine mismatch (C:U and C:C) of Helix II, the known variant of ultra-conserved AGGA motif on the 5′ side of Helix III is indicated by an ellipse and areas of relative conservation between *P. marina* and *P. guyana* are boxed. CBCs between *P. guyana* and *P. marina* are shaded gray across the entire base pair. HCBCs between *P. guyana* and *P. marina* are shaded only on the changed base of the base pair.

Folding of ITS2 rRNA transcripts revealed two very different secondary structures in *Paralia marina* and *P. guyana* with five helices in the former and four in the latter (Figures 11 and 12). Nonetheless, they both demonstrated the hallmarks of ITS2 structures seen in many eukaryotes such as Helix II being relatively short and harbouring a pyrimidine-pyrimidine mismatch; here different for each species with C:U in *P. marina* and U:C and C:C in *P. guyana* (depending on genodeme, arrowheads in Figures 11 and 12). Helix III was the longest and carried known variants of the ultra-conserved motif at the distal end of the 5΄ side of the Helix (UGGU for *P. marina* and AGGA for *P. guyana*, surrounded by ellipses in Figures 11 and 12). These two *Paralia* species also showed other, albeit very few, areas of conservation (boxes in Figures 11 and 12). These included: (1) a six nucleotide sequence which preceded Helix I, (2) five basal pairs of Helix I, (3) part of the spacer between Helix I and Helix II, (4) two basal pairs of Helix II, (5) two base pairs near the terminal loop in Helix II, (6) two base pairs near the base of Helix III, (7) a base pair immediately below the super-conserved motif in Helix III, and (8) the basal part of Helix IV which contained three base pairs. Some of these areas were also conserved between the two species discussed here and species in the *P. longispina* species-complex [6] and some other algae [49,50].

#### Relationship between the taxa examined

**Figure 13:**
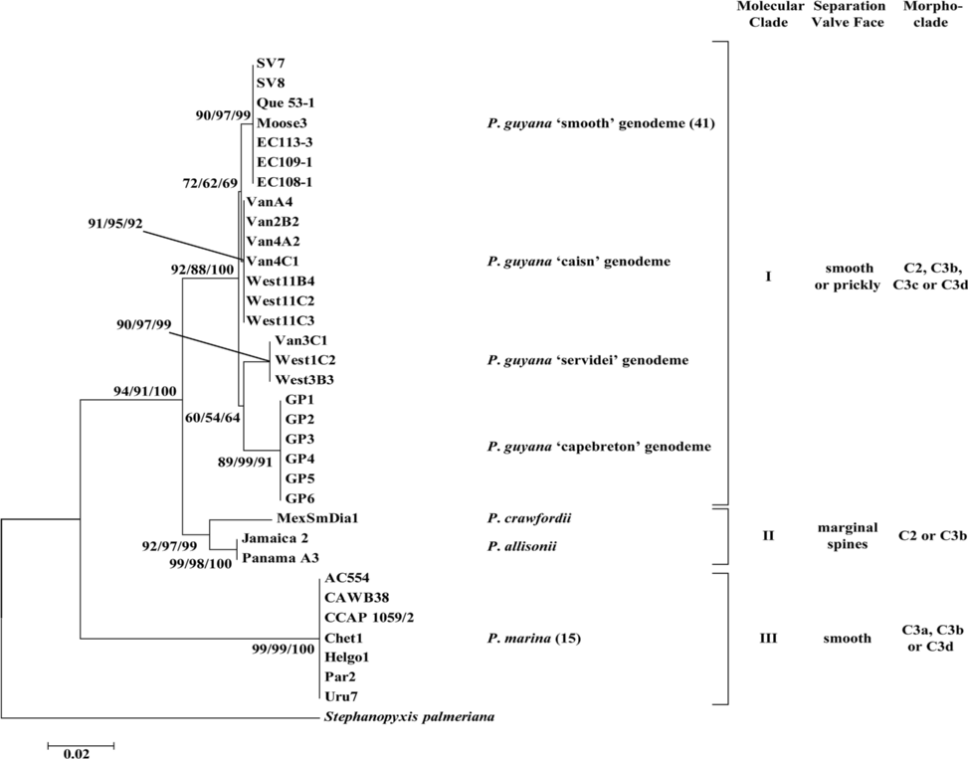
**Maximum likelihood (ML) tree of concatenated, nuclear-encoded 18S and 5.8S + ITS2 sequence of** ***Paralia.*** The concatenated sequence consists of a segment of the 18S gene and a 5.8S + ITS 2 barcode region (*Stephanopyxis palmeriana* is the outgroup; taxa aggregate into three groups, clades I, II and III with bootstrap support (ML/MP/NJ). Separation valve type for each clade is denoted as per Figure 10. The number in parentheses, next to the taxon name represents the number of clones used in construction of the tree if not all are shown (i.e., *P. guyana* ‘smooth’ genodeme and *P. marina*). Representative clones were selected based on methods used to generate Figure 10 (see text for explanation).

**Figure 14:**
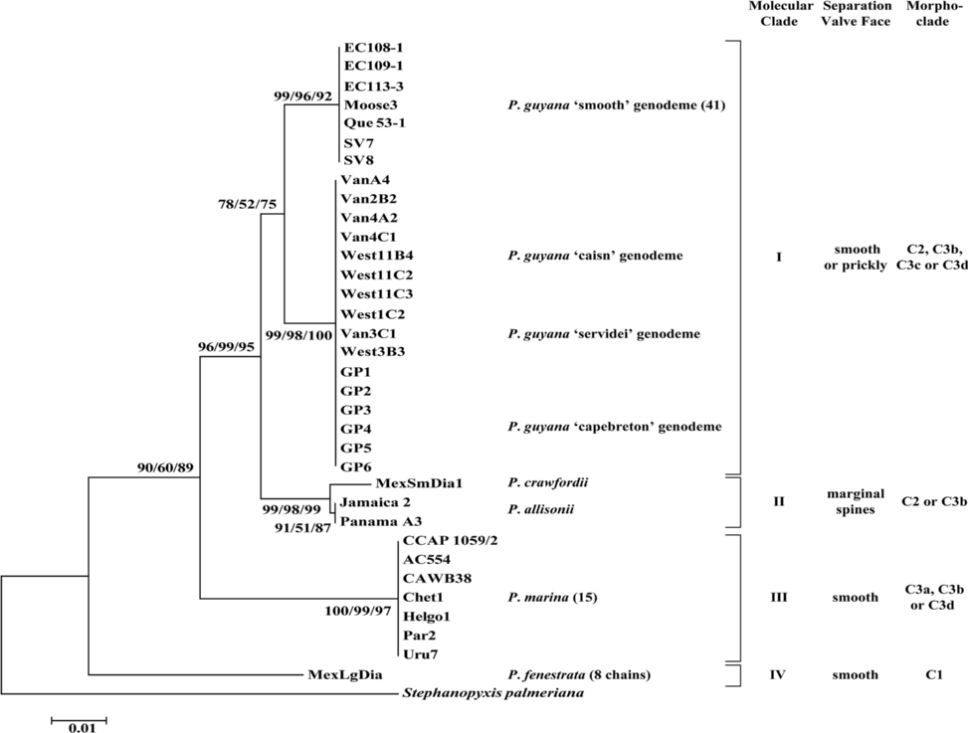
**Maximum likelihood (ML) tree of plastidal-encoded** ***rbc*** **L sequence of** ***Paralia.*** The fragment used corresponded to a 540 bp barcode segment of the RuBisCo large subunit (*rbc*L). *Stephanopyxis palmeriana* is the outgroup; four clades of taxa are labelled I, II, III and IV with bootstrap support (ML/MP/NJ). Separation valve type is shown to the right of the terminal branches. The number in parentheses next to the taxon name represents the number of clones (or single chains in the case of *P. fenestrata*) used in construction of the tree if not all are shown (i.e., *P. guyana* ‘smooth’ genodeme and *P. marina*). Representative clones were selected based on methods used to generate Figure 10 (see text for explanation).

The overall topologies of the trees inferred from all the analyses (ML, MP and NJ) were similar for the 18S, ITS, concatenated nuclear 18S + 5.8S + ITS2 and *rbc*L marker phylogenies. Consequently, we present only the concatenated nuclear encoded (Figure 13) and plastidal *rbc*L trees (Figure 14). In the phylogenetic analysis of 18S + 5.8S + ITS2 three major clades were recovered with similar morphological separation as that found in the *rbc*L (containing larger number of species). The *P. guyana* clade (clade I) received stronger bootstrap support in 18S + 5.8S + ITS2 (92/88/100%, Figure 13) than in the *rbc*L tree (78/52/75%, Figure 14). Each genodeme of *P. guyana* occupied its own terminal branch in the 18S + 5.8S + ITS2 (>89%) tree whereas in the *rbc*L tree, the *P. guyana* ‘smooth’ genodeme and the three prickly genodemes of *P. guyana* formed common terminal groups (>92%). Support was similar for clade II in the concatenated rDNA (94/91/100%) and *rbc*L (96/99/95%) trees. For clade III, comprised of *P. marina*, similar support was shown in the 18S + 5.8S + ITS2 and *rbc*L trees (>97%). *Paralia allisonii* and *P. crawfordii* occupied terminal branches in clade II and had high support in both the concatenated (>92%) and *rbc*L (>98%) trees. The 18S + 5.8S + ITS2 sequence for *P. fenestrata* could not be obtained from the few single chains available; these chains were also uncultivable and so this species is absent from the concatenated tree. Nonetheless, *P. fenestrata* occupied a terminal branch in the *rbc*L tree and received poor to strong support (90/60/89%, Figure 14), depending on the analysis.

## Discussion

### Segregation of taxa

The intercalary valves of the two newly circumscribed taxa (*Paralia marina*, *P. guyana*) and four genodemes of *P. guyana* (‘smooth’, ‘caisn’, ‘capebreton’ and ‘servidei’) were metrically inseparable irrespective of the microscopical methods applied (Table 2). This was well reflected in the results of hierarchical clustering where *P. marina* and all genodemes of *P. guyana* were intermixed in Clade 3. Interestingly, this approach successfully segregates *Paralia longispina*-like species [6]. The external surface structures of separation valves in *P. guyana s.l*. set some, but not all these entities apart. *Paralia marina* and the ‘smooth’ genodeme of *P. guyana* were morphologically cryptic, both in intercalary valve morphometrics and in having smooth separation valve faces. Similar in size, small-diameter separation valves of *P. sulcata s.s*. (when/if such are discovered), may also fall into this category. On the other hand, the three other genodemes of *P. guyana* (‘caisn’, ‘capebreton’ and ‘servidei’) carried prickles or stout spines of various size and abundance on separation valves, but these were only visible on SEM images.

In contrast to morphology, all *P. guyana* demes were genetically distinct from each other and from *P. marina*, but divergence level varied depending on the marker. Not surprisingly, the sequence region with the least divergence was the conservative 373-bp fragment of the 18S gene; all genodemes of *P. guyana* diverged from *P. marina* by d = 0.02. This is twice as much as morphologically distinct *P. allisonii* and *P. crawfordii*, who differ by only 2-bp (or d = 0.01, [6]). In comparison, several morphologically distinct species of *Aulacoseira* Thwaites, the only other genus of the non-polar centrics (Coscinodiscophyceae, [51]) where congeneric sequence data are available for comparison, yield uncorrected d-distances between the species of only 0.01 [52]. In raphid pennate diatoms, such as reproductively isolated species of *Sellaphora* Mereschowsky, sequence divergence in this region is even smaller (d = 0.005), supporting our conclusion that *P. marina* and *P. guyana* are separate species. All genodemes of *P. guyana* showed 100% sequence identity in this gene region.

More divergent sequences were recovered from the 5.8S + ITS2 barcode fragment *sensu* [47,48] and in the equally tested fragment of *rbc*L *sensu* [53] where the inter-/intraspecific guidance thresholds are proposed at d = 0.07 and d = 0.01, respectively, for polar diatoms (centric and pennates); comparisons for non-polar Coscinodiscophycean species are unavailable. Compared to these thresholds, *P. marina* was very distant from *P. guyana* (d > 0.24 for 5.8S + ITS2; d > 0.05 for *rbc*L). The divergence level between the genodemes of *P. guyana* fell within intraspecific thresholds for the 5.8S + ITS2 (d < 0.04). For the *rbc*L fragment, comparisons between the ‘caisn’ and ‘smooth’ genodemes and the ‘capebreton’ and ‘servidei’ genodemes of *P. guyana* fell within proposed intraspecific thresholds (d = 0.01 and d = 0.00, respectively), whereas the ‘capebreton’ and ‘servidei’ genodemes both diverged from the ‘caisn’ and ‘smooth’ genodemes by d = 0.02 which is greater than the proposed intraspecific threshold (d = 0.01). However, MacGillivary & Kaczmarska [6] demonstrated the polar centric DNA barcode threshold for the *rbc*L fragment of d = 0.01 was not infallible as it only pooled 81% of intraspecific sequences compared. Furthermore, the intra-/interspecific *rbc*L DNA barcode threshold may differ between families and even genera ([6], Table 2), reflecting their own rates of evolution.

The secondary structure of the ITS2 transcript was used here as a proxy for reproductive isolation [54-56] or lack thereof [57]. This approach has already been successfully applied to cryptic and semi-cryptic diatom species complexes among raphid e.g., [4,58,59] and araphid pennates [8] and non-polar centrics [6]. The ITS2 secondary structure of *P. marina* (Figure 11) was clearly structurally distinct from *P. guyana* (Figure 12), *P. allisonii* and *P. crawfordii* (figure 45 in [6]); the former had five helices and the latter three species had four and only very few areas of conservation on the four common helices. This is similar to the case of some species of *Sellaphora* [60] where even in relatively closely related congeners sequence alignment of the ITS region is a challenging task. Between-species comparisons of ITS2 secondary structures, following the phylogenetic approach of Caisová *et al*. [49,50] also revealed indels and mutations. The most unanticipated intragenerically were mutations in the YGGY super-conserved motif on the 5′ side of Helix III; UGGU in *P. marina*, AGGU in *P. allisonii* and *P. crawfordii* and AGGA in *P. guyana*, thus far unprecedented among diatoms.

Coleman [55] demonstrated that the presence of even one CBC in conserved regions of Helix II and Helix III coincided with sexual incompatibility between various species of Volvocales. This is also likely true for other green algal orders, albeit mating data are more limited for them, as is the case for a number of algal taxa in general. In Ulvales [49], Chaetophorales, Oedogoniales and some Sphaeropleales [50] CBCs were most commonly found on deeper (not terminal) branches of their phylogenetic trees. In these positions, the CBCs correspond to species-complexes, genera or even higher taxonomic ranks [49,50] rather than species and thus, support the assumption of genetic isolation between members of different CBC-clades is even stronger. Therefore, clear structural differences between the ITS2 secondary structures of all helices in the species discussed here support our conclusion that they represent separate species.

### *Comparison to other morpho-species of* Paralia

The *P. guyana* ‘caisn’, ‘capebreton’ and ‘servidei’ genodemes are the first *Paralia* specimens to demonstrate prickles on the separation valve face. Protrusions on the separation valve face are also present in extinct *P. siberica* (Schmidt) Crawford and Sims, but are more pronounced and round-ended [11]. Additionally, the marginal linking spines are much more robust in *P. siberica* than in the prickly genodemes of *P. guyana* and consist of double spines [11] rather than the single spines present in *P. guyana*.

In contrast to the prickly separation valve faces in the three genodemes of *P. guyana* presented here, the smooth separation valve face, such as in *P. marina* and the *P. guyana* ‘smooth’ genodeme is the most commonly reported morphotype in the literature in both contemporary and fossil samples. For example, *P. sulcata s.l*. valves from natural samples presented in [15] and [12], from Plymouth, UK and various sites near Japan are morphologically inseparable from *P. marina* and from the *P. guyana* ‘smooth’ genodeme in 10 metric characters we examined. Similarly, a recently described Miocene morphospecies, *P. obscura*, found to co-occur with *P. sulcata s.s*. in Ehrenberg’s [16] original material from Oran [10] cannot be set apart from the two taxa examined here. Such a degree of morphological similarity between species, some geologically separated by ~20 MY (i.e., *P. obscura*, *P. marina* and the *P. guyana* ‘smooth’ genodeme), has led to nomenclatural and identification challenges. This must have and will likely continue to lead to ecological incongruities (discussed below) when species recognition is based exclusively on morphological characters on the intercalary valves in particular. Our results, therefore, illustrate the usefulness of molecular tools in *Paralia* taxonomy, especially in recognising the ‘smooth’ morphotype. The level of sequence divergence in ITS2 found between *P. marina* and the *P. guyana* ‘smooth’ genodeme, uncommon among diatom congeners thus far examined, suggests the significant potential for the existence of other undiscovered, morphologically cryptic species in this complex.

### Taxonomic implications for ecology

*Paralia sulcata s.l*. is commonly reported worldwide and from a wide range of environments. Morphologically cryptic and semi-cryptic diversity discovered here within this “species” may help to reconcile conflicting reports of *P. sulcata s.l.* ecology. For example, studies have shown that some populations of *P. sulcata s.l*. have competitive advantage and thrive under low light conditions [61-63], but other reports have indicated others who have an advantage when irradiance levels are high, such as during bright winter days [64]. Interestingly, the studies by Margalef [61], Zong [62] and McQuoid & Nordberg [63], all occur in Europe and the Hobson & McQuoid [64] work involved *Paralia sulcata s.l*. populations from British Columbia, Canada. Our data suggest that these two sets of studies may have been in fact conducted on two different species of *Paralia*. The species of *Paralia* we reported from western Canada were not found (in our study) in Europe and *vice versa*. A similar case may be made for nitrogen optima as McQuoid & Nordberg [63] noted that relative abundance of *P. sulcata s.l*. among surface sediments in western Sweden is positively correlated with the degree of mixing in the water column, but negatively correlated with higher seawater nitrogen concentrations. Conversely, Liu *et al*. [65] found greater abundance of *P. sulcata s.l*. in waters which are richer in nitrogen. Since the study by McQuoid & Nordberg [63] was conducted in Sweden (Atlantic Ocean) and the study by Liu *et al*. [65] occurred in the South China Sea (Pacific), those studies may have also been, in fact, carried out on two discrete, physiologically different species of *Paralia*. As our data illustrate, the availability of globally consistent tools to recognise investigated species (when valve morphology may be insufficiently differentiated) is a necessary prerequisite to better understand the autoecology, including biogeography, of diatoms.

### Biogeography

Undoubtedly, the genus *Paralia* Heiberg is cosmopolitan. However, some species complexes within this genus may not be. For example, based on what is known about their distribution to date, the morphologically distinct *P. longispina*-like species and specimens (see [6]) were all thus far recovered from subtropical and tropical locations between 17°50′S (Viti Levu, Fiji) and 26°38′N (Haha-jima, Japan); none were recovered from temperate, shallow coastal waters of North America despite extensive sampling. Conversely, *P. guyana* and *P. marina* were recovered only from more temperate and eutrophic waters. *Paralia guyana* was found on both the Atlantic and Pacific coasts of North America; its distribution ranged as far north as Botwood, Canada (49°08′N) and as far south as Marshall, USA (38°09′N). Samples from both the east and west coasts of Canada contained the ‘smooth’ genodeme of *P. guyana* whereas the prickly genodemes had a more restricted distribution with one genodeme, ‘capebreton’, found at only one site (Grove’s Point, Nova Scotia), despite intensive sampling of the Canadian Maritimes. In contrast, *Paralia marina* had a much wider distribution as it was recovered from the European Atlantic, and single sites in Uruguay, New Zealand, and eastern Canada. Similar types of intrageneric distribution, with some taxa being restricted while others cosmopolitan, may be found, for example, among *Navicula* Bory de Saint-Vincent [4] or *Asterionellopsis* Round [8]. These and our work may represent a distribution pattern that will be seen more commonly [9] as the application of molecular methods in diatom taxonomy and consequently in ecology (e.g., using high throughput sequencing) becomes more frequent. For such studies molecular delineation of diatom species is of great significance.

Although *P. marina* specimens examined were widely distributed, sequences of all three markers examined in this study (*rbc*L, 18S and ITS) show 100% identity. The maintenance of such a degree of sequence identity by benthic diatom populations from oceans worldwide may be due, at least in part, to its capacity to survive transoceanic voyages in ship ballast waters and sediments. Chains of *Paralia* were reported from nearly every ship ballast examined e.g., [23-25]. *Paralia* species have demonstrated the ability to survive weeks in ship ballast tanks; both in the water column (up to 14 days in this study and 33 days elsewhere [24]) and possibly even longer in ship ballast sediments [36]. Therefore, ship-mediated dispersal may contribute to the capacity of some *Paralia* species (exemplified by *P. marina*) to cross their natural barriers and maintain genetic homogeneity worldwide.

### Paralia in TAVs

*Paralia marina* was the only representative of the genus recovered from ship ballast tanks from the trans-Atlantic voyages examined here and comprised 96% of the cells recovered from the three TAVs. Additionally, *P. marina* cell concentrations at the end of each voyage, and after mid-ocean ballast water exchange, ranged from 13 cells l^−1^ in TAV1 to 540 cells l^−1^ in TAV3. These quantities were comparable to those reported from Pacific crossings for *P. sulcata s.l.* by Klein *et al*. ([24]; 5–28 cells l^−1^) and Dickman & Zhang ([21]; 5–40 cells l^−1^ L]. *Paralia marina* cell concentrations at the end of TAV3 were the second highest cited in the literature to date, next to *Paralia* specimens enumerated in Ruiz & Smith ([66]; 18000 cells l^−1^). Given the known tank volume, the average *Paralia marina* specific propagule size in one ballast tank at the end of each of the three TAVs would be $\overline{x}$ =130592 cells. If the 5.47 × 10^9^ litres of ballast water arriving in eastern Canada from Europe in 2006–2007 [67] is a typical annual volume arriving and in position to legally deballast, the number of cells of *P. marina* these ships carry per year may be estimated at 7.14 × 10^14^ cells. This is a large propagule size, particularly when de-ballasting concentrates in just a few ports (or areas) and continues for decades as it has in Atlantic Canada. Thus, it is unanticipated to discover *P. marina* in only one site in eastern Canada (Cheticamp, Nova Scotia) despite extensive sampling for *Paralia* throughout the region. Lockwood *et al*. [34] and Simberloff [35] argued that a large propagule size and multiple propagule introductions are two of the best predictors of the successful establishment of a species in a non-native territory, but of course other factors may interfere. However, the same conditions do not generally correlate with secondary dispersal from the site of primary establishment. It therefore would be worthwhile to conduct an additional survey to determine if the species has spread since our collection in 2009.

Secondary dispersal, involves other factors (natural and/or anthropogenic), as evidenced by numerous examples of failed intentional introductions [68-70]. Some of these factors may involve suboptimal physico-chemical conditions in the species’ non-native environment; e.g., temperature, salinity and nutrients. These might play, at least in part, a role in the case for *P. marina* de-ballasted in eastern Canadian waters. The physico-chemical regimes present on the Canadian side of the Atlantic may be sufficiently fitting to sustain, but not to expand *P. marina* populations. For example, Gebühr *et al*. [71] investigated the seasonality of *P. marina* (clones Helgo1, Helgo2, Helgo3, Helgo4; reported as *P. sulcata* in [71]) and found that temperature, light and nutrient conditions were the environmental factors with the strongest effect on their abundance. Furthermore, although similar in some properties, the coastal waters of the European and Canadian Atlantic differ in a number of specific physico-chemical characters with Europe having, for example, generally greater annual average sea-surface temperatures [72], salinities [73], nutrient (e.g., nitrogen and silica, [74]) and dissolved oxygen [75] concentrations. This does not mean that *P. marina* may not ever establish a stronger presence in eastern Canadian waters in the future if strong propagule pressure continues. In some cases a long lag time exists between species introduction and species establishment [76,77] and longer still before the species expands beyond its original establishment site. In addition, species which have been outside their physico-chemical optima may become capable of establishing pioneer populations in non-native habitats because of favourable changes brought on by global climate change or by favourable mutations in the founder population [35].

## Conclusions

Examination of the taxa presented here and earlier [6,10,12] shows the existence of three morphotypes defined by their separation valve face morphology. The *P. longispina* group (*P. allisonii*, *P. crawfordii*, *P. ehrmanii* and *P. longispina*) has marginal triangular spines on separation valves. The *P. guyana* (genodemes ‘caisn’, ‘capebreton’ and ‘servidei’) which often have prickles on the separation valve face constitute a second group. The *P. marina* group (*P. marina*, the ‘smooth’ genodeme of *P. guyana*, *P. obscura*, *P. sulcata s.s*. and *P. fenestrata*) has smooth separation valve faces. This latter group may be further subdivided into *P. sulcata*-like species (*P. sulcata s.s*. and *P. fenestrata*) with pronounced fenestrae and *P. marina*, *P. guyana* ‘smooth’ and *P. obscura*, with subtle fenestrae often overlaid by a siliceous lid, thus obscured in SEM (examining the valve surface), but visible in the light (transmitting) microscope. Each of these groups consists of multiple, generally morphologically semi-cryptic or cryptic species.

Genetic delineation, on the other hand, segregates all these species and their demes readily. Correct identification of species is at the heart of species and community ecology, including non-indigenous introductions. We demonstrated that despite the long and strong propagule pressure of *P. marina* arriving in transoceanic ship ballast tank waters from European sites, it was only found in one site in Eastern Canada in 2009. We also demonstrated that ship ballast sediments arriving to Eastern Canadian ports from intercoastal locations contain live *Paralia* chains, which, when rejuvenated in laboratory cultures all proved to be the ‘smooth’ genodeme of *P. guyana*. We also demonstrated that all specimens of *Paralia* arriving at Canadian coasts in ballast of trans-Atlantic vessels examined are *P. marina*. This suggests that dispersal opportunity is not the barrier for *P. marina* for establishing stronger presence on the Atlantic coast of Canada. Because the two species specimens are cryptic and semi-cryptic, we thus advocate for the wide use of molecular means of species recognition in monitoring the establishment and further, secondary dispersal of *Paralia* species outside their native geographical ranges. This is of particular significance in eastern Canada where the propagule pressure of *P. marina* continues and the change in the local environment, man-mediated or otherwise, may facilitate the permanent colonization of this diatom in this region.

## Methods

### Establishment of monoclonal cultures

Seawater and sediment samples were collected from 45 inter- and subtidal sites and from ship ballast sediments; five in Europe, one in New Zealand, one in Uruguay and 38 from the Atlantic (including 15 ship ballast sediments) and Pacific coasts of North America. Only the last port of call is identified for ballast sediments (Table 1 and discussed detail in Villac & Kaczmarska [36] and Villac *et al*. [25]). A total of 184 clones were initially established as described in MacGillivary & Kaczmarska [6]. These were screened morphologically and genetically; the widest range of cell diameter and 100% identity in genetic markers were criteria for selection of the final set of 76 monoclonal cultures included in detailed analyses (Table 1). All culture isolates were grown in f/2 media at 20°C at an irradiance of 40–50 μmol m^−2^ s^−1^, to facilitate a “common garden” experimental condition with the aim to emphasise genetic (in contrast to specific for individual local environmental) aspects of frustule morphology.

### Ballast water on board processing, cell counts and molecular identification

In addition, ballast water was sampled from ships during three trans-Atlantic voyages (TAVs). TAV1 (October 15–24, 2008) and TAV2 (November 15–24, 2008) crossed from Rotterdam, Netherlands to Sept-Îles, Quebec, Canada. TAV3, from September 16-October 2, 2009, originated in Bar, Montenegro and ended in Havre-Saint-Pierre, Quebec, Canada. One ballast tank on each ship was sampled at the beginning and the end of the voyage, and at least once during the crossing. Sampling protocol and diatom enumeration in the samples from the three TAVs followed Klein *et al*. [24] except here the entire contents of the 3-8 L of collected ballast water sample was filtered, settled and examined due to the exceptionally low abundance of diatoms in these samples. Diatoms from TAVs 1, 2 and 3 were counted from tank 5S, 7S and 6P (S = starboard and P = port), respectively on voyage day 9, 8 and 14, respectively. Cells were considered live at the time of collection and only counted when intact chloroplasts auto-fluoresced. Following cell counts, the ITS region of 18 *Paralia* chains were amplified following [78] with the following exceptions: immediately after isolation into PCR tubes, cells were subjected to three freeze/thaw cycles at temperatures of +/−80°C to break heavily silicified frustules and liberate DNA and the annealing temperature of primers in the PCR cycle was adjusted to 50°C.

### DNA extraction, amplification and sequencing

DNA of cultured cells was extracted from frozen *Paralia* pellets harvested in mid-exponential growth phase using an UltraClean Soil DNA Kit (MoBio Laboratories, Carlsbad, CA, USA) following manufacturer’s recommendations. Three different sequence regions were amplified. From nuclear ribosomal RNA, a portion of the small subunit (18S) region (373 bp long, 195 bp downstream from the start codon) and a 682–711 bp internal transcribed spacer (ITS) region were amplified using primers 18sF and 18sR [79] and ITS1 and ITS4 [80] and *Paralia*-specific primers designed for this study: ParFOR1 (5′-GCCTGTGAAATAGCCTCCT-3′), ParFOR2 (5′-CCGACAATAGGGTGA-CCTG-3′), ParREV1 (5′-GCGGGTATTCTTACTTAACTTG-3′) and ParREV2 (5′-CCGGACTC AAACCAACAAG-3′), respectively. The fragment (540 bp long, 417 bp from the start codon) of the plastidal-encoded large subunit of Rubisco (*rbc*L) was amplified using primers DtrbcL2R ([81]; as NDrbcL8) and DtrbcL2F, DtrbcL3F and DtrbcL3R [78]. The sequence regions targeted were amplified and sequenced following MacGillivary & Kaczmarska [6,53] and edited and aligned with BioEdit version 7.0.5.3 [82] and Clustal W version 1.4 [83]. The ITS2 portion of the ITS region was aligned between taxa with the aid of ITS2 secondary structures.

### Sequence analysis

Molecular grouping of clones into respective species and genodemes was demonstrated in the form of trees. These were constructed from a concatenated sequence consisting of the nuclear 18S rRNA fragment and the 5.8S + ITS2 barcode [47,48] and from a *rbc*L sequence representing a supplemental DNA barcode for diatoms [53]. All trees were constructed using MEGA version 5.03 [84]. Distance (neighbour-joining, NJ) trees were inferred from the pairwise Kimura-2-Parameter (K2P) model. Maximum parsimony (MP) trees were generated by close-neighbour-interchange (CNI) on random trees. For Maximum Likelihood (ML) trees, jModeltest version 0.1.1 [85] in Bosque [86] was used to select optimal base substitution models according to Akaike Information Criterion (AIC). The Hasegawa, Kishino and Yano (HKY) + Gamma (G) model (−ln L = 1033.68) and the Generalised time-reversible (GTR) + G (−ln L = 1531.00) were the most optimal for the nuclear and the *rbc*L sequence fragments, respectively. Confidence of branching was calculated using 10000 bootstrap replicates for each tree. Clones included in both trees matched those included for HCA. Published sequences for members of the *P. longispina* species complex [6] and *P. fenestrata* (for *rbc*L only) were also included in analyses (Table 1). *Stephanopyxis palmeriana* (Greville) Grunow was used as an outgroup for all trees, based on the close relationship between *Paralia* and *Stephanopyxis* in multigene phylogenies i.e., [87].

### ITS2 secondary structure analysis

ITS2 secondary structure models were constructed using mfold version 2.3 ([88], http://mfold.rna.albany.edu/?q=mfold/RNA-Folding-Form2.3) using default parameters except for temperature, which was set to 20°C to reflect the culture growth environment. Structures were drawn using VARNA 3.1 ([89], http://varna.lri.fr). Helices were labelled according to Mai & Coleman [90]. Specific structures were compared following the phylogenetic approach of Caisová *et al*. [49,50].

### Acquisition and analysis of morphometric data

At least 10 randomly-encountered, cleaned valves from each of the monoclonal cultures were subjected to SEM-based ([91] or [92], as appropriate) morphometric analysis. The average of the metrics from each of 10 valve characters (1–3, 5, 7–11 in Figure 1 and pervalvar axis length, as in [6,10,12,15]) were used in a hierarchical clustering analysis (HCA). For this analysis, where genetically delineated taxa and genodemes (a group of conspecific individuals differing from others genetically [93]) were represented by eight or more clones (i.e., *P. marina* and *P. guyana* ‘smooth’ genodeme), numbers were randomly assigned to clones and a random number generator was used to select which seven clones would be included in HCA analysis. This was done to preserve figure clarity. HCA was performed using pvclust [94] running 10000 multiscale bootstraps and giving approximately unbiased [95,96] *p*-values of branch point probabilities in the dendrogram. Morphological terminology follows [6,10-12,15]; terms ‘relief’ and ‘intaglio’ refer to both external and internal linking spines in intercalary valves while only to internal linking spines in separation valves.

### Examination of preparation BM1021

Valves of *Paralia* from W. Smith’s preparation BM1021 from the Greville collection, British Natural History Museum, were examined using LM to better understand Smith’s [17] original concept of *Paralia marina* (as *Orthosira marina* in that publication). Only the 40× objective could be used to examine these specimens because of the excessive thickness of the slide. Consequently, only the coarse and taxonomically non-informative morphological characters could be observed (i.e., diameter, fenestrae and marginal linking spines), but they were nonetheless considered in general comparisons. Un-mounted material from W. Smith’s original gathering (if in existence) was not available to the authors.

### Accession numbers

[GenBank: ITS: KP150089-KP150160, KP193457, JN201575, JN201577, JN201579; 18S: AY485227, KP149947-KP150016, JN201583, JN201585, JN201587; *rbc*L: KP150017-KP150088, KP253080, JN201591, JN201593, JN201595].

## Additional file

Additional file 1:
**Sequence differences in the ITS2 (above) and 540 bp** ***rbc*** **L fragment (below) between the four genodemes of** ***P. guyana*** **.** For ITS2 the positions of the variable sites are indicated in terms of base number in the ITS2 sequence and which helix they occur. The number of variable sites is tallied above the sequence differences. HCBCs between *P. guyana* ‘servidei’ genodeme and all other genodemes of *P. guyana* are shaded in gray. For *rbc*L sequences only the base number position and number of variable sites are presented. A ‘.’ indicates 100% identity between the sequences.

## Abbreviations

AIC: Akaike information criterion
AU: Approximately unbiased
BGBM: Botanical gardens and botanical museum
BOLD: Barcode of life database
CBC: Compensatory base change
CCAP: Culture collection of algae and protozoa
CNI: Close-neighbour-interchange
G: Gamma
GTR: Generalised time-reversible
HCA: Hierarchical clustering analysis
HCBC: Hemi-Compensatory Base Change
HKY: Hasegawa, Kishino and Yano
ITS: Internal transcribed spacer
K2P: Kimura-2-parameter
ML: Maximum likelihood
MP: Maximum parsimony
NJ: Neighbour-joining
*rbc*L: RuBisCo large subunit
TAV: Trans-Atlantic voyage
