# Supplementary figures and images for: Paralia (Bacillariophyta) stowaways in ship ballast: implications for biogeography and diversity of the genus
Source: J Biol Res (Thessalon). 2015 Feb 15;22(1):2. doi: 10.1186/s40709-015-0024-5 (PMC4389653; doi:10.1186/s40709-015-0024-5)

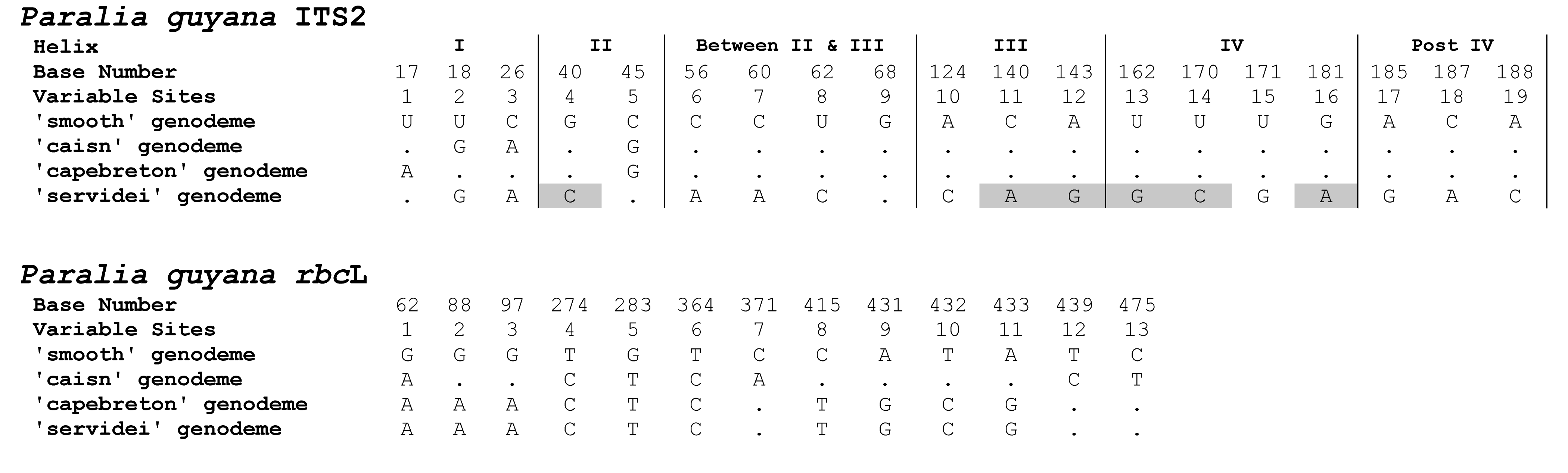

Supplement: Additional file 1: — Sequence differences in the ITS2 (above) and 540 bp rbc L fragment (below) between the four genodemes of P. guyana . For ITS2 the positions of the variable sites are indicated in terms of base number in the ITS2 sequence and which helix they occur. The number of variable sites is tallied above the sequence differences. HCBCs between P. guyana ‘servidei’ genodeme and all other genodemes of P. guyana are shaded in gray. For rbcL sequences only the base number position and number of variable sites are presented. A ‘.’ indicates 100% identity between the sequences. [file 40709_2015_24_MOESM1_ESM.tiff]
